# Revisiting Sub-Band Gap Emission Mechanism in 2D Halide Perovskites: The Role of Defect States

**DOI:** 10.1021/jacs.4c06621

**Published:** 2024-08-08

**Authors:** Igal Levine, Dorothee Menzel, Artem Musiienko, Rowan MacQueen, Natalia Romano, Manuel Vasquez-Montoya, Eva Unger, Carlos Mora Perez, Aaron Forde, Amanda J. Neukirch, Lars Korte, Thomas Dittrich

**Affiliations:** † Helmholtz-Zentrum Berlin für Materialien und Energie GmbH, Division Solar Energy, Kekuléstraße 5, 12489 Berlin, Germany; ‡ Institute of Chemistry and The Center for Nanoscience and Nanotechnology, The Hebrew University, Jerusalem 91904, Israel; § Theoretical Physics and chemistry of Materials, Los Alamos National Laboratory, Los Alamos, New Mexico 87545, United States; ∥ Department of Chemistry, University of Southern California, Los Angeles, California 90089, United States

## Abstract

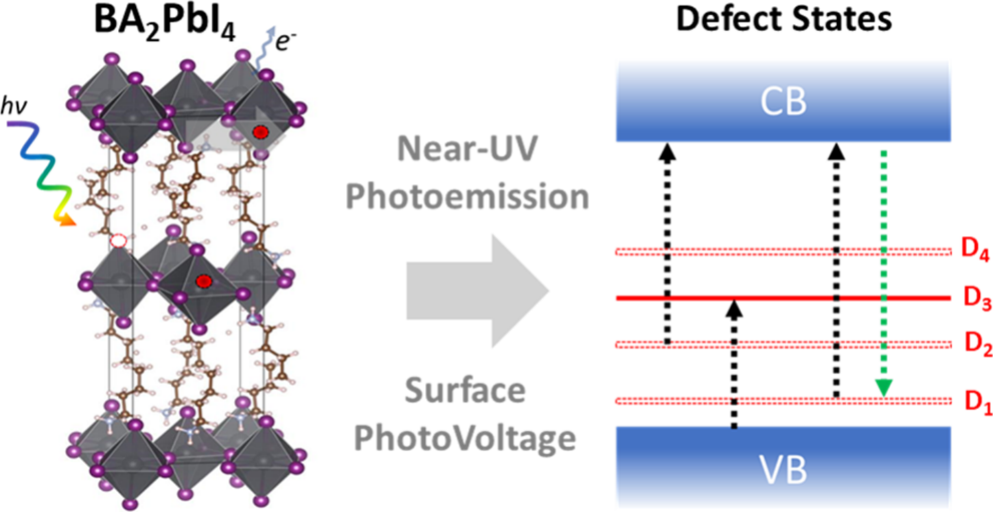

Understanding the sub-band gap luminescence in Ruddlesden–Popper 2D metal halide hybrid perovskites (2D HaPs) is essential for efficient charge injection and collection in optoelectronic devices. Still, its origins are still under debate with respect to the role of self-trapped excitons or radiative recombination via defect states. In this study, we characterized charge separation, recombination, and transport in single crystals, exfoliated layers, and polycrystalline thin films of butylammonium lead iodide (BA_2_PbI_4_), one of the most prominent 2D HaPs. We combined complementary defect- and exciton-sensitive methods such as photoluminescence (PL) spectroscopy, modulated and time-resolved surface photovoltage (SPV) spectroscopy, constant final state photoelectron yield spectroscopy (CFSYS), and constant light-induced magneto transport (CLIMAT), to demonstrate striking differences between charge separation induced by dissociation of excitons and by excitation of mobile charge carriers from defect states. Our results suggest that the broad sub-band gap emission in BA_2_PbI_4_ and other 2D HaPs is caused by radiative recombination via defect states (shallow as well as midgap states) rather than self-trapped excitons. Density functional theory (DFT) results show that common defects can readily occur and produce an energetic profile that agrees well with the experimental results. The DFT results suggest that the formation of iodine interstitials is the initial process leading to degradation, responsible for the emergence of midgap states, and that defect engineering will play a key role in enhancing the optoelectronic properties of 2D HaPs in the future.

## Introduction

Dion-Jacobson and Ruddlesden–Popper layered (“quasi-2D”) hybrid perovskites (hereinafter termed simply as “2D HaPs”) have emerged as promising semiconductors with tunable emission suitable for a variety of device applications.^1,2^ Furthermore, 2D HaPs are being introduced as passivation layers for devices based on 3D HaPs, enhancing their efficiency and stability.^3−5^ One of the typical features that 2D HaPs exhibit is sub-band gap photoluminescence (PL). Understanding the origin of sub-band gap photoluminescence (PL) emission in semiconductor materials is crucial for their development as active layers in optoelectronic devices such as LEDs and solar cells. Such sub-band gap emission hinders precise control over the desired emission wavelength and the resulting LED performance, and is a signature of additional charge recombination paths that result in a loss in the photovoltaic efficiency of solar cell devices. As a typical sub-band gap emission mechanism, relaxation of photogenerated carriers to defect states within the band gap and subsequent radiative recombination induces light emission with photon energies below the band gap. An alternative mechanism for sub-band gap PL emission, is the self-trapped exciton (STE) mechanism, which has often been suggested.^6,7^ Depending on the underlaying mechanism, photoexcited charge carriers can either contribute to photocurrent through the material or alternatively stay as bound excitons. Distinguishing between these two mechanisms can elucidate whether the sub-band gap emission is an inherent property of the material (STE), or it can be completely mitigated by removal of the defects responsible for it.

**Figure 1:**
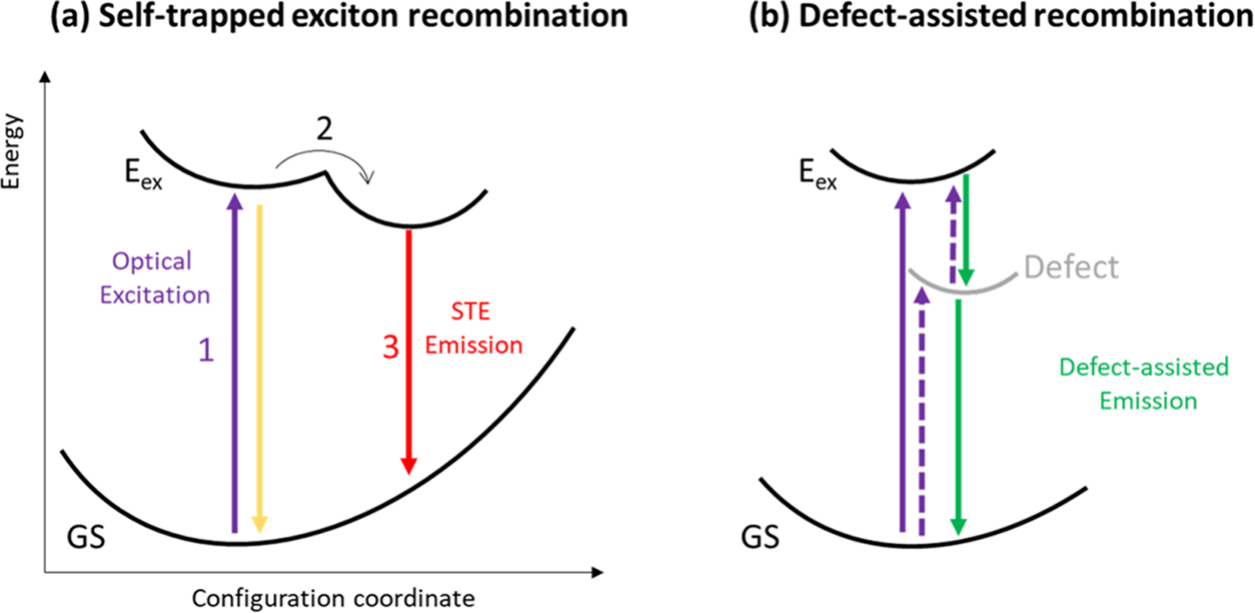
Diagrams describing the self-trapped exciton (STE) mechanism (a) vs defect-assisted recombination mechanism (b) Purple, yellow, green, and red arrows denote optical excitations, band-to-band radiative decay transition, defect-assisted radiative decay transitions and radiative decay transitions via self-trapped excitons, respectively. The numbering in (a) denotes the temporal sequence of the STE process: (1): fundamental excitation into the exciton state (E_ex_) from the ground state (GS); (2) structural deformation due to strong electron–phonon coupling; (3) below-band gap STE emission.

The two different sub-band gap emission mechanisms are described schematically in Figure 1. In the STE mechanism (Figure 1a), strong electron–phonon coupling is required, in which upon above band gap (supra-band gap) photoexcitation and the formation of excitons, a new “deformed” excited state with reduced energy forms from which sub-band gap photons can be emitted. Previously, such mechanism has been suggested to be responsible for sub-band gap emission in metal halides and rare gas crystals.^8^ Specifically for metal halides, it was proposed that the initial event leading to STE formation is actually the localization of the photogenerated hole in the newly formed excited state, generating a self-trapped hole, followed by bounding of an electron to the self-trapped hole site, resulting in an STE.^8^ For decades, STE has been suggested to explain the emission properties of various metal halides,^8,9^ metal oxides with an ultrawide band gap (i.e., >5 eV)^10,11^ and some metal oxides with a wide band gap such as titania (anatase).^12^

In spite of its higher complexity compared to defect-assisted recombination, the STE mechanism has become quite popular in recent years to explain sub-band gap emission in Dion-Jacobson and Ruddlesden–Popper layered hybrid perovskites.^7,13−15^ However, recent studies question this assignment, and there is growing evidence that defect-assisted recombination as illustrated in Figure 1b is the more likely cause for the sub-band gap emission in this family of materials.^16−22^

STE requires fundamental absorption from the ground state to the excited state to form an exciton. Therefore, it is possible to distinguish between STE and defect-assisted emission by comparing sub-band gap PL emission feature(s) with corresponding feature(s) in the UV–vis absorption spectrum, or, alternatively, by using sub-band gap excitation in the acquisition of the PL emission spectrum, as was successfully shown by Hu et al.^23^ and Kahmann et al.^19^ It is known that halide perovskites exhibit a high degree of electron–phonon coupling, due to their ionic and soft lattice nature, resulting in polaron formation.^24^ In addition, it was shown that for some HaP compositions defects are present, with sub-band gap transition energies.^25,26^ Thus, another possibility arises in which STE and defects coexist, where the excitons get trapped at the defect sites, resulting in defect-assisted STE recombination.^27^

To date, all studies used methods based on optical properties of 2D HaPs, such as PL and UV–vis absorption, as the main characterization tools. A drawback for interpretation of PL spectra is the difficulty in assigning the dominant recombination mechanism (free carriers/bound excitons/trapped carriers). One of the key differences between defect-assisted and STE mechanisms is the difference in transport properties of photogenerated species involved. In a defect-assisted mechanism, remaining untrapped mobile charge carriers can diffuse or drift and therefore contribute to electrical currents. Excitons and STEs are neutral quasi-particles which do not contribute to electrical currents. However, dissociation of excitons into mobile charge carriers can induce additional electrical currents.

In our work, we use this fundamental difference of contributions to electrical currents in order to distinguish experimentally between defect-related and STE mechanisms. For this purpose, we combine methods characterizing sub-band gap emission by radiative and photoelectron emission (PL and CFSYS – Constant Final State Photoelectron Yield Spectroscopy,^28^ respectively) with methods characterizing separation of photogenerated charge carriers in space (transient and modulated SPV – Surface PhotoVoltage – Spectroscopy^29^) and (photo)electrical currents and mobilities (Hall and photo-Hall,^30^ or CLIMAT – Constant Light Induced Magneto Transport). We showed in previous studies of 3D HaPs that SPV and CFSYS are highly sensitive contactless methods that directly detect defect states in 3D HaPs whereas these defects could not be detected by PL and other optical methods.^28,31,32^

SPV signals are generated when photogenerated charges are being spatially separated. In general, the sign of an SPV signal is determined by the direction of charge separation and the resulting net difference in the (average) location of positive and negative charges. A net larger hole density near the surface would result in a positive SPV signal, and vice versa. Light-Modulated SPV spectroscopy measurements rely on absorption and not only significantly enhance the S/N, but also allow to distinguish between SPV signals that follow the light modulation vs retarded SPV signals.^29^ Transient SPV (tr-SPV) probes the charge carrier dynamics over more than 7 orders of magnitude in time after photoexcitation with a short (ns) light pulse.^33^ The fundamental difference between charge transport mechanisms in STE and defect-assisted recombination induces two radically different pictures of charge separation detected by our optoelectronic methods.

Experiments were performed on single crystals, peeled layers and polycrystalline thin films of 2D butyl ammonium lead iodide (BA_2_PbI_4_), a typical 2D HaP, in order to show the general behavior of electronic transitions in relation to the mechanisms of charge separation. It is shown that BA_2_PbI_4_ is a p-type semiconductor with a very low dark equilibrium carrier concentration, indicating that the majority of trapped carriers are electrons. Dissociation of free excitons caused electron trapping at defect states at/near the surface. In contrast, excitation at photon energies below and above the energy of the free exciton transition resulted in preferential separation of photogenerated holes toward the surface. We find several shallow and deep defect states that are evidenced by independent measurement techniques, and show that the broad sub band gap emission in 2D HaPs is caused by defects, not by STE. To elucidate the chemical nature of the defects, we then perform ab initio electronic structure calculations that show that common, readily formed defects in a BA_2_PbI slab lead to trap states similar in nature to those we experimentally observe. Furthermore, we demonstrate that during photoemission measurements, 2D HaPs readily undergo degradation and deep defects close to midgap are formed, which, using the calculation results, are found to be iodine (I) interstitials.

## Results and Discussion

### Shallow Defects

**Figure 2:**
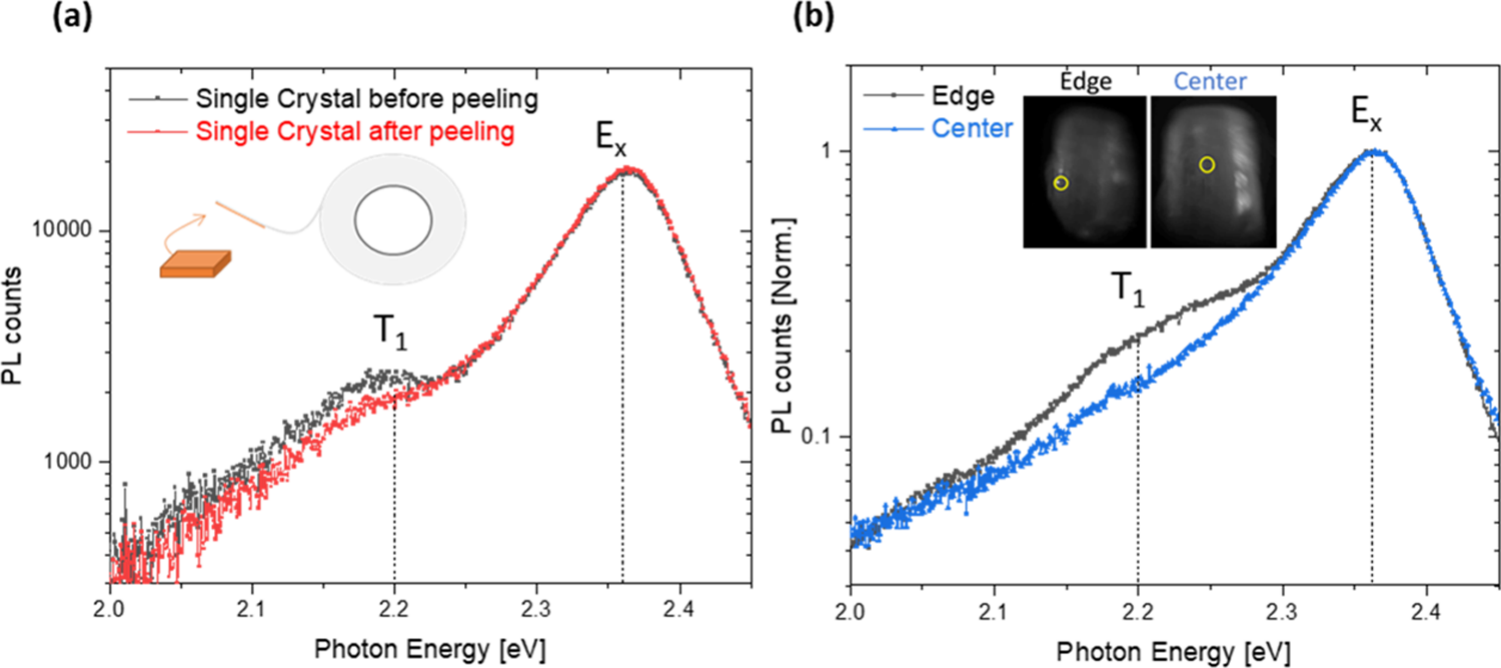
PL emission spectra of a BA_2_PbI_4_ single crystal: (a) before and after peeling with a 3 M scotch tape on a semilogarithmic scale; (b) at the center of the crystal (blue) and on the edge (black), normalized. Dashed lines are shown at *E* = 2.20 eV (T_1_ transition) and *E* = 2.36 eV (exciton emission, *E*_*x*_). The excitation wavelength was 472 nm, at a fluence of ∼7 × 10^13^ photons per cm^2^.

Typical PL spectra of a BA_2_PbI_4_ single crystal before and after peeling are shown in Figure 2a. The observed peak at 2.36 eV matches well with the excitonic band gap emission at room temperature reported in the literature.^34^ Interestingly, an additional emission peak below the band gap is observed at about 2.2 eV (termed hereinafter as transition T_1_). This sub-band gap feature at 2.2 eV has been initially claimed to be caused by a “second band gap”.^35^ However, in a following study, the same authors concluded that this feature was actually related to Pb–I interlayer interaction, which is enhanced at the crystal edges.^36^ As can be seen from Figure 2a, upon peeling off the top layer of the crystal, the relative PL intensity of the shoulder decreases. In addition, when comparing the PL spectra at the edge vs the center of the peeled crystal, the relative PL intensity at the same energy region (2.2 eV) is much higher on the edge of the crystal (Figure 2b). Hence, these findings raise the question whether this feature at 2.2 eV is an intrinsic property of the material, or related to surface/edge defects.

In order to gain further insight into the nature of the potentially defect-related PL emission at 2.2 eV, modulated SPV measurements were performed on a freshly peeled BA_2_PbI_4_ single crystal.

**Figure 3:**
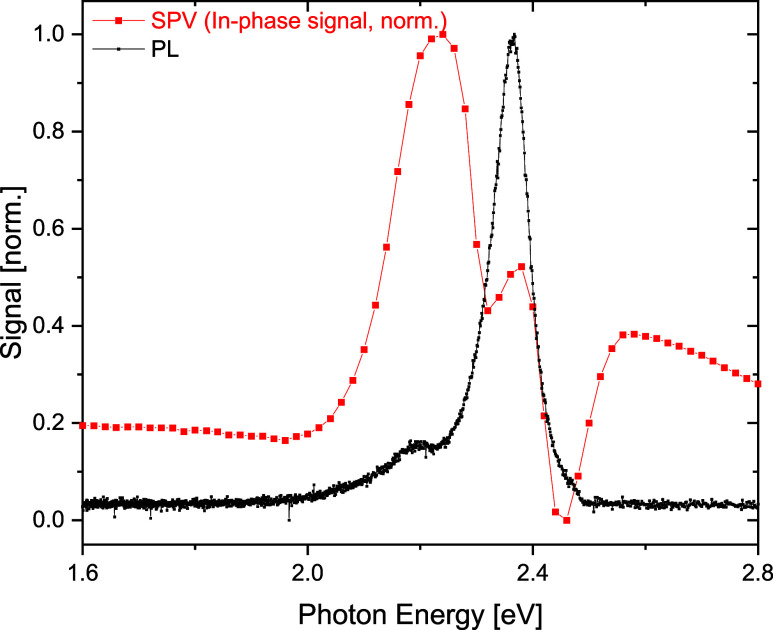
PL emission spectra (black) vs the in-phase signal of the modulated SPV measurement result (red) of a BA_2_PbI_4_ single crystal.

A comparison between the in-phase component of the modulated SPV signal (the fast component that follows the light modulation period^29^) and the PL spectrum is shown in Figure 3 (For a reference UV–vis spectrum of a flake of the single crystal, see Figure S1). Interestingly, while both the sub-band gap and excitonic features are seen clearly in both measurements, the trend in their relative intensities is opposite. In contrast to the PL spectrum, where the feature at 2.2 eV appears as a shoulder to the main peak centered at 2.36 eV, in the SPV spectrum, the signal at 2.2 eV is much larger than that at 2.36 eV. This finding is explained by the different nature of the two methods: while PL is more bulk-sensitive, the SPV signal can be very sensitive to the surface,^29,37^ and hence any surface or edge-related defects that contribute to charge separation processes would result in a larger SPV signal compared to the main excitonic feature. Since SPV relies on charge separation, i.e., the motion of free photogenerated charge carriers, the higher signal at 2.2 eV directly excludes the formation of STEs, since an excitation energy of 2.2 eV is not sufficient in order to generate excitons in the first place. As evident from the SPV results, where in contrast to PL, a signal could be observed only when charge separation occurs, these photogenerated free carriers readily undergo charge separation at the crystal edges (horizontal surface/grain boundaries), which will be discussed later in the text. In addition, a non-negligible SPV signal is observed below 2 eV which is attributed to absorption related to deep defects. Since in this regime below the band gap straylight effects can play a significant role, an appropriate long-pass filter needs to be used, as shown in Figure S2.

In Figure 3, an additional rise of the in-phase SPV signal is observed at higher photon energies, which can be attributed to the band-to-band transition at about 2.55 eV. Thus, from our SPV measurements we find that the exciton binding energy for the BA_2_PbI_4_ amounts to about 190 meV (2.55–2.36 eV), which lies within the wide range of exciton binding energies reported in literature ranging from values as low as 80 meV (based on UV–vis and PL)^34^ to values of about 300 meV (based on photoelectron spectroscopy).^38^ Furthermore, a dip between the excitonic transition and the band-to-band transition is observed around 2.4 eV. Such a change in the magnitude of the in-phase signal hints to a change in the charge separation mechanism (i.e., a variation of the e–h separation direction) around the exciton transition. This point will be investigated in more detail by transient SPV (tr-SPV) spectroscopy in the following section.

### Charge Separation Kinetics and Deep Defects

**Figure 4:**
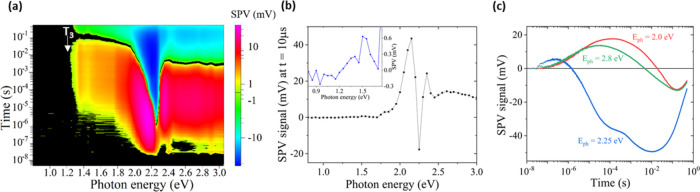
Contour plot of time-resolved SPV spectroscopy of a BA_2_PbI_4_ single crystal (a); Transient SPV spectrum, deduced at *t* = 10 μs, including a closeup at low photon energies in the inset (b); and (c) selected transients at different laser excitation energies: 2.0 eV (red), 2.25 eV (blue) and 2.8 eV (green).

Figure 4a shows a contour plot, i.e., the map of the SPV signals as a function of photon energy and logarithmic time, of the time-resolved SPV spectrum of an as-peeled BA_2_PbI_4_ single crystal. Two main features are seen from Figure 4a: First, the onset of the SPV signal starts at photon energies as low as ca. 1.2 eV (transition T_3_), i.e., roughly around midgap, providing direct experimental evidence for the existence of deep defects close to midgap. Second, a change of the sign from positive to negative SPV signal occurs, where the change of the sign occurs at much longer times (ms range) for photon energies below and above the band gap but at much shorter times (ns···μs range) for photon energies around the free exciton transition. The similar times at which the sign changes below and above the free exciton transition suggest a similar charge separation mechanism for excitation at both these photon energies.

Figure 4b shows an SPV spectrum deduced at 10 μs after the start of the excitation pulse, including a closeup at the low photon energy regime shown in the inset. The onset of positive SPV signals at 1.2 eV can be clearly seen. To confirm that the observed onset at 1.2 eV does not originate from experimental artifacts such as variations in the laser intensity as a function of wavelength (which were suppressed using a variable beam expander, as shown in Figure S3) or processes related to 2-photon absorption (which are highly unlikely due to the relatively long pulses (2–3 ns) used in the tr-SPV measurements), sub-band gap modulated SPV spectra were recorded using halogen lamp with a 610 nm long pass filter (to suppress straylight), and a similar SPV onset at ca. 1.2 eV was observed, as shown in Figure S2 in the SI. Around 1.8 eV, a strong increase of the positive SPV signals set on. Between 2.15 and 2.2 eV, the SPV signals decrease strongly and change to maximum negative signal at *E*_phot_ = 2.25 eV. In the following, the SPV signal changes to positive sign for photon energies up to about 2.35 eV. To conclude, the formation of free excitons causes a change of the direction of charge separation, in contrast to other transitions.

**Figure 5:**
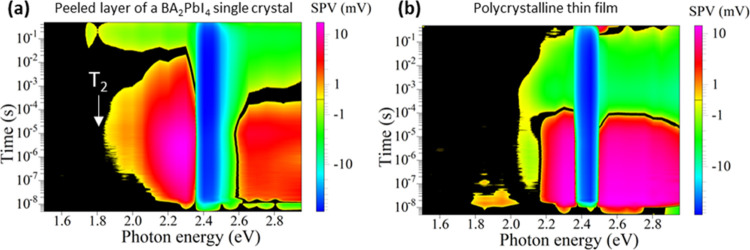
Contour plots of time-resolved SPV spectroscopy of a peeled layer of a BA_2_PbI_4_ single crystal on carbon tape (a); and of a polycrystalline thin film deposited on ITO (b).

The different quality in charge separation for excitation below, within and above the energy of free exciton generation can be also seen in the transients depicted in Figure 4c. The transients excited at 2.0 and 2.8 eV are very similar to a monotonous increase of the SPV signals in time up to a maximum at about 30 and 150 μs, respectively, and with a monotonous decrease toward negative signals with a minimum at about 0.16 s. In general, we find negligible dependence of the transients on excitation energies above the band gap (Figure S4), as expected, since the excited higher-energy carriers quickly cool down to the band edge (within sub-ps to ps). In contrast, the decay of the transient excited at 2.25 eV reaches its minimum at about 10 ms, which is preceded by well pronounced bump of a positive signal between 30 and 500 μs. The existence of the bump in the decay of the transient excited at 2.25 eV gives evidence for the superposition of several processes of charge separation and relaxation that are occurring simultaneously. The fact that a sub-band gap SPV signal is observed already from photon energies as low as 1.2 eV, suggests a substantial density of deep defect states (no excitons are formed at energies below 2.2 eV). The behavior of sub-band gap SPV transients is very different to these excited around 2.25 eV, i.e., within the transition of free excitons. Therefore, the observed sub-band gap SPV signals provide direct experimental proof that sub-band gap transitions, including PL emission, can be directly related to defect-assisted processes such as defect-assisted recombination in case of PL emission. In other words, the observations of sub-band gap SPV signals, alongside with the fact that no excitons are formed below 2.25 eV, suggest that the STE model can be ruled out for the 2D HaP BA_2_PbI_4_ single crystal. For comparison, Figure 5 shows the contour plots of peeled BA_2_PbI_4_ thin layer on carbon tape (a), and a polycrystalline thin film of BA_2_PbI_4_ spin coated from solution onto ITO (b). Both contour plots are qualitatively very similar to that measured for the BA_2_PbI_4_ single crystal, with three significant differences: (i) The negative signals, which are typical for the photon energy range leading to free exciton formation, have their onset already at the shortest times, i.e., within the duration time of the laser pulses (5 ns), (for selected transients at different laser excitation energies see Figure S5) and suggest fast dissociation of excitons, in line with other works.^39−41^ (ii) The center of the negative signals shifted toward about 2.45 eV. (iii) The SPV signals set on at photon energies of about 1.8 eV (transition T_2_). Therefore, the transition energy of the free excitons is higher by about 0.2 eV for the spatial regions closer to the surface and for thin films of the BA_2_PbI_4_ a in comparison to the BA_2_PbI_4_ single crystal. Furthermore, charge separation related to excitation from defects close to midgap could not be observed on peeled BA_2_PbI_4_ thin layers and on spin coated BA_2_PbI_4_ thin films. This finding could be related to the spatial distributions of the deep defects, i.e., deep defects related to transition T_3_ could be distributed in the bulk of the BA_2_PbI_4_ single crystal (as also confirmed in the modulated SPV spectra shown in Figure S2 in the SI), or, alternatively, that the different synthesis conditions of the single crystals vs the spin coated polycrystalline thin films result in a lower density of deep defects for the thin films.

Incidentally, the qualitative behavior of SPV signals in the ranges below, around and above the transition of free excitons was also very similar for a slightly different perovskite composition of a PEA_2_PbI_4_ single crystal and PEA_2_PbBr_4_ polycrystalline thin films, as shown in Figure S6 in the SI. This points to the fact that the observed phenomena are not affected by changes in the organic spacer and/or halide anion in the 2D HaP lattice, but are caused by more general mechanisms of charge transport and charge separation in 2D HaPs. Furthermore, to show that our conclusion is more generic and there is no need to invoke the STE model in order to explain sub-band gap emission in other 2D HaPs, we also performed modulated SPV measurements on thin films of PEA_2_PbI_4_, BA_2_PbBr_4_ and PEA_2_PbBr_4_ (see Figure S7). In all cases, we find sub-band gap SPV signals that point to the existence of defect states and charge carrier separation, suggesting that any observed sub-band gap emission can be related to these defect states. It is important to note that the obtained defect-related transitions (i.e., transitions below the transition energy of the free excitons) reflect the bulk of the single crystal, since the optical absorption coefficients for photon energies below the transition energy of the free excitons are orders of magnitude lower than those above it. This implies that sub-band gap absorption, and, therefore, any possible carrier generation/release occurs at least up to a few microns below the surface, and hence mostly reflects the bulk area of the single crystals. However, in the case of strong absorption in the exciton regime and above the band gap, carrier generation would be limited to the first few 100 nm of the surface. Hence, in these regimes, the charge carrier dynamics that we find is mostly related to the surface and first few 100 nm below the surface of the single crystal. Therefore, the tr-SPV dynamics obtained at these energies are highly dependent on surface quality and surface states.

### Mechanisms of Charge Transport and Charge Separation

The qualitatively different behavior of SPV signals excited at photon energies within the transition of free excitons in comparison to SPV signals excited at photon energies within defect related and valence band to conduction band transitions points to the existence of two different mechanisms of charge transport and charge separation in 2D HaPs depending on the excitation.

**Figure 6:**
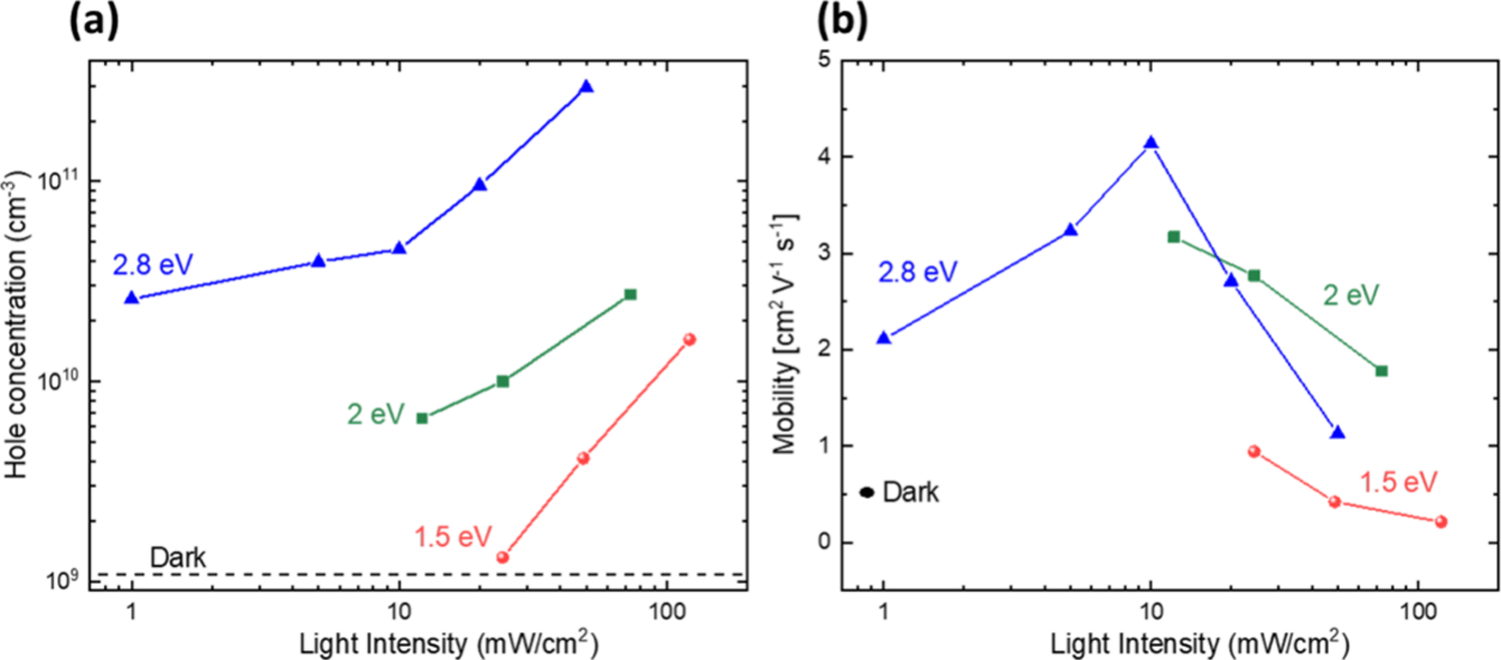
Concentration (a) and mobility (b) of photogenerated holes as a function of light intensity resulting from CLIMAT of a BA_2_PbI_4_ single crystal for excitation at 1.5, 2.0, and 2.8 eV (red circles, green squares and blue triangles, respectively). The dashed line in (a) and the black ellipse in (b) give the values in the dark.

The polarity of minority and majority charge carriers plays a decisive role regarding trapping of charge carriers. CLIMAT measurements^42^ showed that holes are the majority and electrons are the minority charge carriers in our 2D HaPs. The concentration of holes was very small in the dark and amounted to only about 10^9^ cm^–3^ and increased to more than 10^11^ cm^–3^ under illumination depending on light intensity and photon energy (see Figure 6a). Furthermore, the hole mobilities ranged between 0.5 cm^2^/(V s) (dark, excitation of holes from deep defects) and 4 cm^2^/(V s) (band-to-band excitation at 10 mW/cm^2^, see Figure 6b). The corresponding diffusion constants of holes amount to 0.012 and 0.1 cm^2^/s. For comparison, the diffusion constant of excitons is significantly higher, in the order of 1 cm^2^/s.^39^ Overall, the mobilities of holes (0.5–4 cm^2^/(V s)) are in very good agreement with literature values, such as those obtained via optical pump–THz probe measurements for PEA_2_PbI_4_ (1 cm^2^/(V s)^43^ and 7.6 cm^2^/(V s)^44^) and BA_2_PbI_4_ (3.4 cm^2^/(V s)),^44^ as well as the mobility that was obtained via time-resolved microwave conductivity (TRMC) for BA_2_PbI_4_ (0.3–0.4 cm^2^/(V s)).^45^ Still, these mobilities are low in comparison to the mobilities of conventional semiconductors such as crystalline silicon (electron mobility of 1350 cm^2^/(V s))^46^ but of the same order as for amorphous silicon (of the order of 1 cm^2^/(V s)).^47^ Therefore, it appears that potential fluctuations that could be caused, for example, by defects and/or band discontinuities between 2D domains, limit the transport of charge carriers in 2D HaPs.

**Figure 7:**
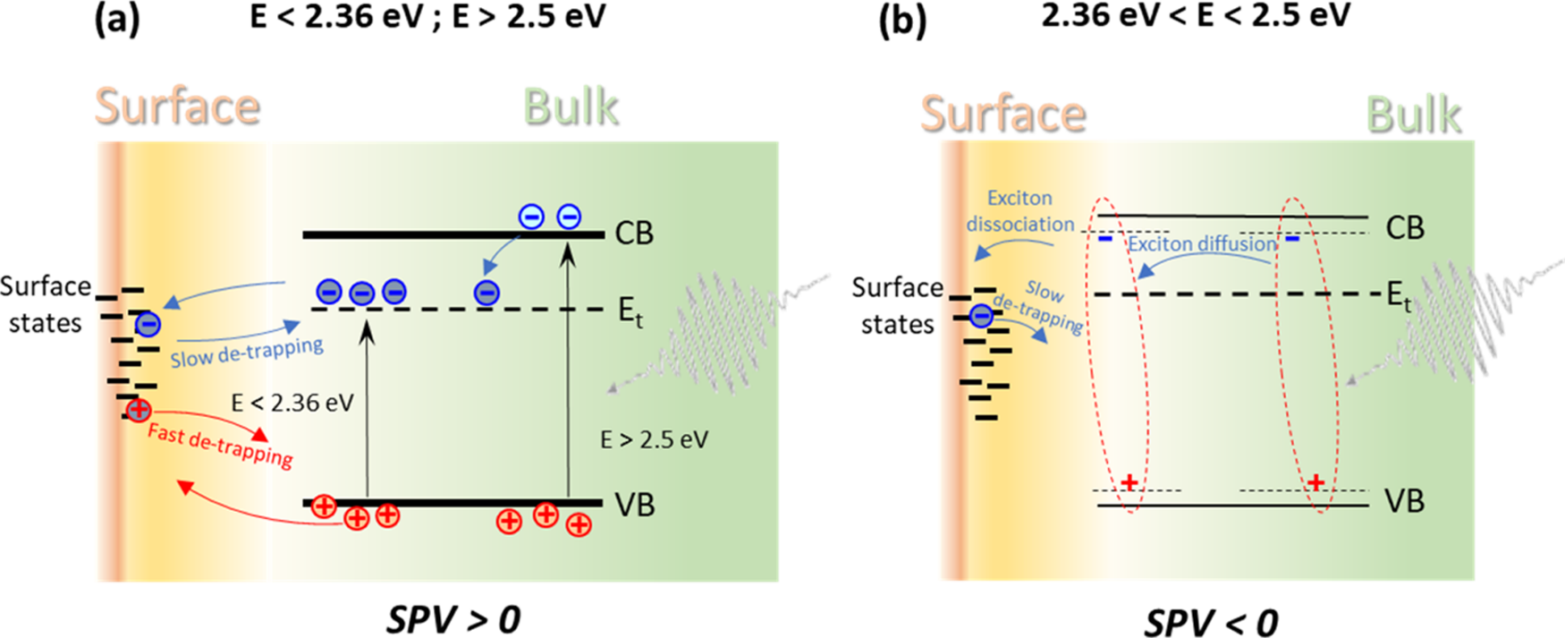
Simplified band diagrams explaining the dominating mechanisms of charge separation for excitation in the ranges below and above the excitonic regime (a) and in the range of the excitonic regime (b). The transfer of photogenerated excitons toward the surface is faster than the transfer of holes and much faster than the transfer of electrons toward the surface. The detrapping of electrons trapped at surface states is much slower than the detrapping of holes trapped at surface states. *E*_t_ denotes a representative defect level, for a more detailed defect distribution map, see Figure 9.

The qualitative behavior of charge separation and relaxation was similar for single crystals, peeled layers and thin films of 2D HaPs. Thus, it appears that charge separation is dominated by the surface and the influence of internal or buried interfaces can be neglected. As a consequence, we surmise that the mechanisms of charge separation are based on separation of charge carriers (Figure 7a) and on dissociation of excitons (Figure 7b) at surface states.

It is reasonable to assume that surface states are available for both electrons and holes and that in dark equilibrium, electron traps are less saturated than hole traps in the bulk of 2D HaPs (remark: the term “trap” is used by keeping also in mind the possibility of potential discontinuities at interfaces between 2D domains). Under these assumptions, upon illumination it is more probable that photogenerated electrons get trapped in the bulk. Therefore, more photogenerated holes than photogenerated electrons will reach the surface within shorter times and can be trapped there at surface states. The preferential trapping of photogenerated holes at surface states results in positive SPV signals at shorter times. At longer times, electrons can also reach the surface and holes trapped at surface states can be detrapped faster than electrons trapped at surface states. This causes a change of the sign of SPV signals from positive to negative.

The change of the sign of the SPV from positive to negative was observed for excitation below and above the transition energy of free excitons, i.e., in the defect range, and in the range of band-to-band absorption, respectively. Furthermore, SPV transients behave similarly for excitation via defect transitions and via band-to-band absorption. This means that both electrons and holes can reach the surface at different rates, and, since electrons were found to be the minority carriers, that the majority of traps are electron traps.

The excitation with photon energies around 2.4 eV and subsequent dissociation of excitons at surface states resulted in negative SPV signals. This means that surface states attract much more strongly electrons than holes during the dissociation of excitons. In this sense, excitons serve as an electron shuttle to surface states and exciton diffusion is the dominant transport mechanism of photogenerated charge carriers for excitation around the exciton energy of 2.4 eV.

For peeled layers and thin films of 2D HaPs, the dissociation of excitons started within the laser pulse. The slow relaxation times of electrons trapped at deep surface states, in combination with fast detrapping of holes that diffuse away from the surface, results in negative SPV signals in the range of the exciton transition energy throughout the entire probed time domain.

For the crystal of 2D HaPs, in contrast, the sign of SPV transients changed from positive to negative in the range of the transition of free exciton generation at times between about one μs and one ms. Furthermore, the highest positive SPV signals were observed for the 2D crystal in the range of the transition of free exciton generation. This means that excitons dissociate also in the bulk of crystals of 2D HaPs. It is intriguing that this phenomenon was not observed for peeled layers and thin films of 2D HaPs and that the transition energy of free exciton generation was significantly higher for the peeled layers and thin films of 2D HaPs than for the bulk of crystals of 2D HaPs. It seems that deep defects, which were not observed in peeled layers and thin films of 2D HaPs, are related to the dissociation of free excitons in the bulk of crystals of 2D HaPs.

### Evolution of Defect States

**Figure 8:**
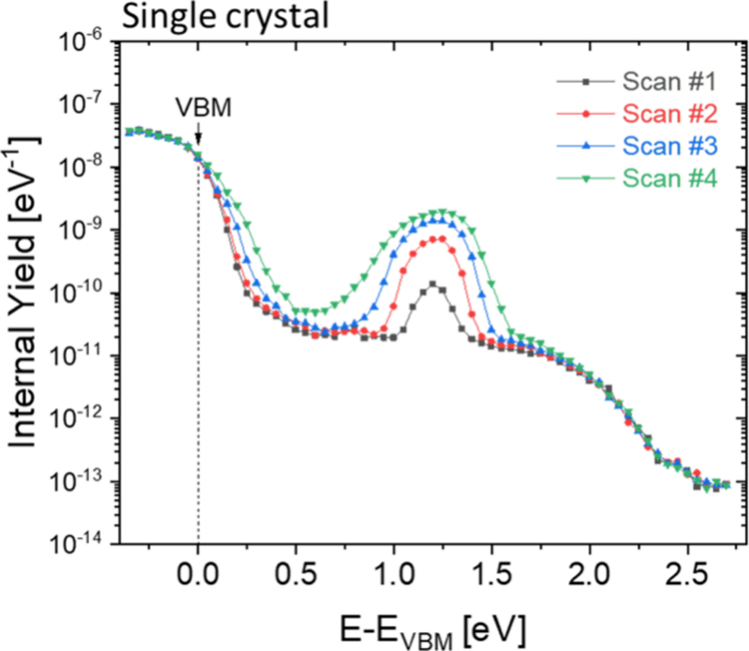
Photoelectron yield spectra of consecutive CFSYS measurements (Scans 1–4) of a BA_2_PbI_4_ single crystal.

To map the defect distribution using a different, independent method, we turn our attention to constant final state photoelectron yield (CFSYS) measurements, which have been proven to be a valuable tool in detecting low densities of occupied defects in 3D HaPs.^28^ The results of CFSYS measurements of a BA_2_PbI_4_ single crystal are shown in Figure 8, as a function of the energetic distance from the valence band maximum (VBM) (for more details on the determination of the VBM see Section S2 in the SI as well as for the CFSYS spectra plotted vs the photon energy/binding energy scale, please see Figure S9). As CFSYS is a photoemission related method, we only observe occupied states, thus below the Fermi level. We find a significant, broad defect-related feature above the VBM, spanning nearly the entire gap, in combination with a less broad, distinct defect feature around 1.2 eV above VBM.

The CFSYS spectra shown in Figure 8 provide additional experimental evidence for the existence of a broad distribution of defect states, in-line with the tr-SPV results shown in Figure 4. It is known that metal halide perovskites can decompose in ultrahigh vacuum.^49,50^ Therefore, the evolution of a very broad observed defect distribution can be explained by the generation of point defects, the formation of a very thin layer of PbI_2_ and/or of precursors of such a layer. We previously found using CFSYS that gap (defect) states are present in PbI_2_ with a broad distribution of about 1.5 eV above the VBM,^28^ which we further confirm using modulated SPV, as shown on Figure S8. Furthermore, ongoing reduction of lead iodide toward elemental lead would contribute to the defect distribution with occupied states up to the Fermi edge, as typically observed for metals. As a remark, since the CFSYS is extremely sensitive, the concentrations of PbI_2_ and elemental lead could be far below the sensitivity of X-ray photoemission spectroscopy or X-ray diffraction.

**Figure 9:**
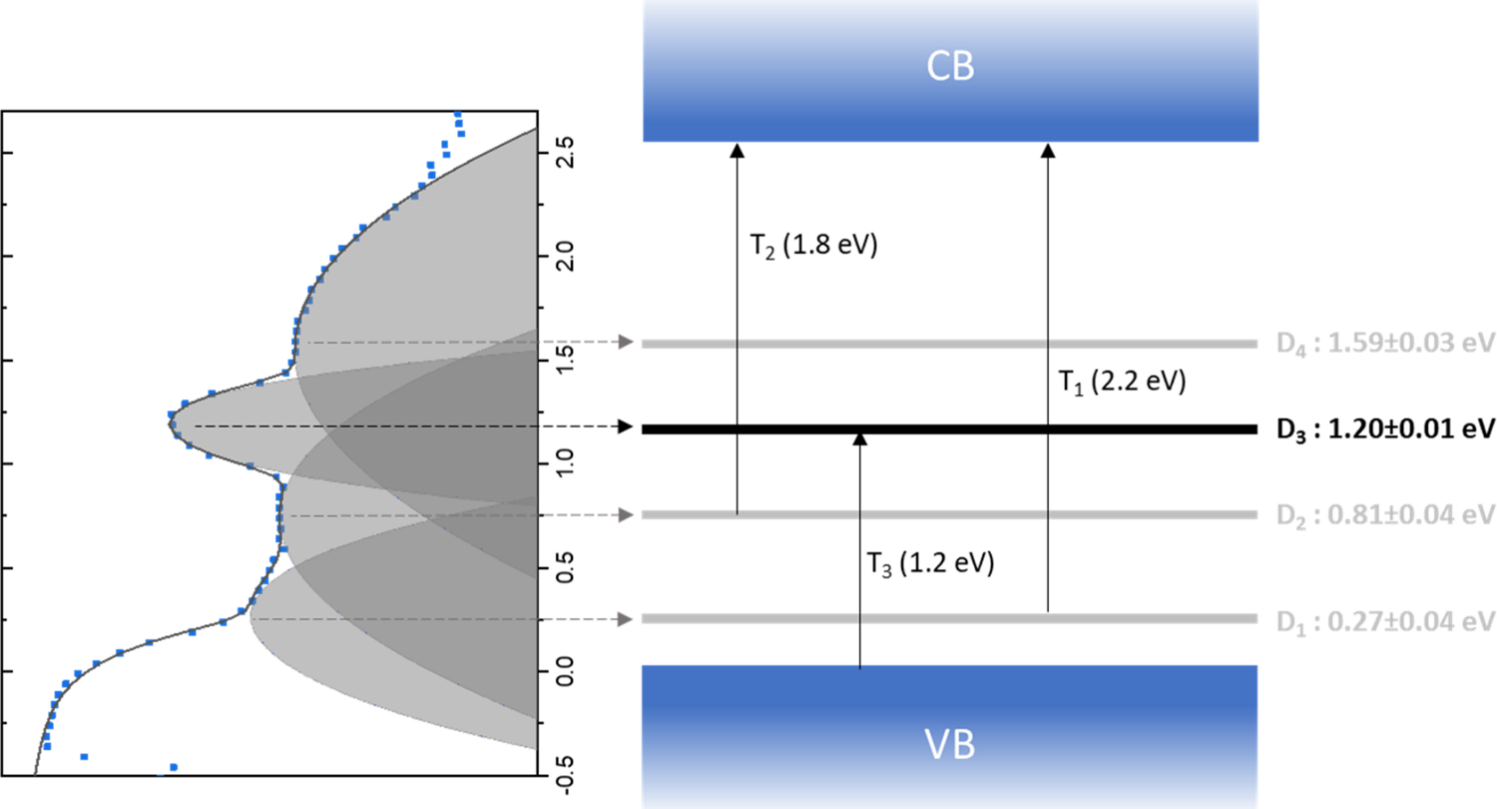
Suggested band diagram mapping the energetic defect distribution in BA_2_PbI_4_, extracted from the deconvolution results of the CFSYS measurements shown on the left. Defect levels D_1_, D_2_ and D_4_ are shown in gray lines, D_3_ is shown in black, transitions from the SPV measurements are shown as black arrows.

We will now explore whether the very broad defect distribution observed in the CFSYS measurements can be linked to the different transitions observed by SPV measurements–where transition energies of 2.2 (T_1_), 1.8 (T_2_) and 1.2 eV (T_3_) were found. Deconvolution results of the defect region in the CFSYS spectra of the single crystal into 4 individual defect positions are shown in Figure S10. Figure 9 summarizes the defect positions w.r.t the VBM (averaged over the 4 different scans, for details see Figure S11a). Interestingly, for the single crystal, a prominent defect peak (D_3_) appears at ca. 1.2 eV above the VBM (5.0–5.2 eV photon energies, as shown in Figure S9). By performing several subsequent scans, we find a measurement-induced defect formation, seen clearly as the rise in intensity of D_3_. This intensity saturates, but the peak continues to broaden with increasing number of scans (See Figure S11b). In addition, from the fitting procedure outlined in Section S2, we find that the exponential band tail parameter (defined as the inverse of the exponential slope of the VB) increases with the number of scans as well, from a low value below the resolution limit of our setup in the first scan, to nearly 50 meV in the fourth scan (see Figure S11c).

For the sake of comparison, a sequence of consecutively measured CFSYS spectra of a polycrystalline thin film is shown in Figure S9 in the SI. For the polycrystalline thin film, the defect peak around midgap (D_3_) is missing in the initial spectrum, giving additional evidence that this defect transition is caused by reactions in UHV in combination with UV illumination. With ongoing CFSYS measurements, or after an XPS measurement, an even more severe beam-induced damage appears for the thin film (Figure S9), including significant defect broadening, alongside with significant increase in the defect densities above the VBM, suggesting X-ray induced beam damage for the polycrystalline thin film.

Based on the deconvolution results and the defect levels obtained from the CFSYS measurements (D_1–4_), a suggested defect band diagram is shown in Figure 9 alongside with possible assignments of the different transitions observed in the SPV measurements (T_1–3_). We note that the fwhm of the fitted Gaussian defect peaks D_1_, D_2_ and D_4_ (shown as gray lines in the energy diagram) is quite large, and hence the exact defect positions still contain a large amount of uncertainty. In contrast, the energetic position of D_3_, (shown in black), is extracted with more confidence due to the much narrower fwhm. It is important to note that in order to try and assign the observed defect levels from the CFSYS to the transitions seen in the SPV measurements, one needs to take into account possible structural reorganization of the lattice when the occupation of a certain defect changes, resulting in a Franck–Condon (FC) shift. Since in the CFSYS measurements, only occupied defects are being probed, the obtained energetic position corresponds to the position after any possible reorganization of the lattice. However, in the case of SPV, which relies on absorption, two possible scenarios are possible: (1) band-to-defect transition; and (2) defect-to-band transition. In (1), the obtained transition extracted from SPV corresponds to a defect position before reorganization, since the defect level was empty prior to the absorption event. However, in (2), since the defect level is occupied, the transition corresponds to the energetic level after reorganization. Thus, comparing between the energetic positions obtained from CFSYS to SPV is not straightforward. In our case, to a first approximation, we first assume that small-to-negligible FC shifts are present in BA_2_PbI_4_, and find relatively good agreement for defects D_1_,D_2_ and D_3_ with transitions T_1_, T_2_ and T_3_, suggesting that indeed our underlying assumption is useful and small-to-negligible FC shifts are present in BA_2_PbI_4_. With regards to D_4_, we could not find a transition that corresponds to this defect level, which could be due to a relatively large FC shift present for this specific defect. Furthermore, care must be taken with regards to D_4_ since its fwhm is larger than 0.5 eV, suggesting an extremely wide defect band as a result of the fitting procedure, which is probably not physical. Also, the CFSYS spectrum shows only the states in the band gap that are occupied by electrons, i.e., states up to the Fermi level *E*_F_. In our measurements it appears as if D_4_ lies well below *E*_F_ (cf. Figure S9b,d). However, this is an artifact due to charging of the samples, which could not be avoided due to experimental constraints. The actual energetic distance *E*_F_ – *E*_VBM_ is about 2.0–2.2 eV. Therefore, D4 is actually close to the cutoff of the spectrum caused by the Fermi occupation function, which further complicates the fitting.

Our results are in line with the charge separation mechanism described in section (b), since the comparison between the PL and the SPV measurements (Figure 3) suggest that the density of D_1_ is greater closer to the surface, and that it is a shallow recombination center for holes, yet a very deep trap for electrons.

**Figure 10:**
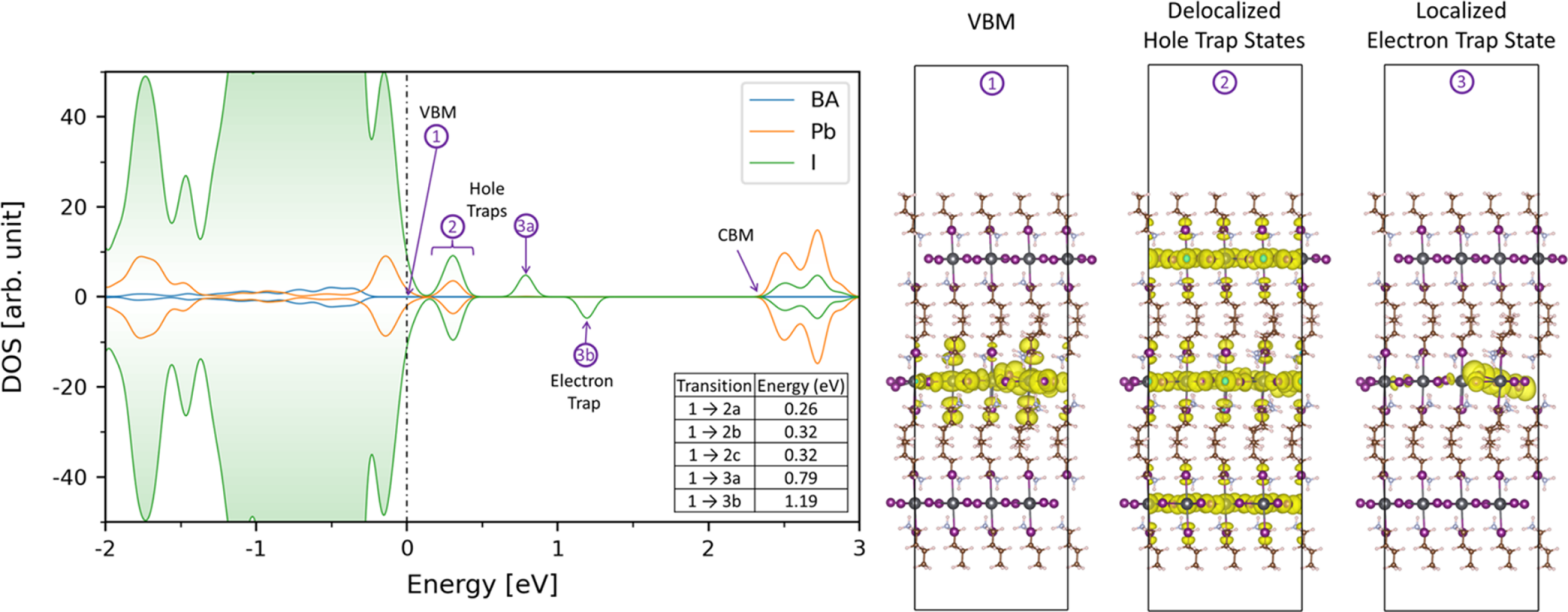
Projected density of states (pDOS) of the “Bulk” like I interstitial (I_I_ 4) system with spin-polarized calculation: BA ligand (blue), Pb (orange), I (green). Along with the band charge density of 1 (Experimental VBM), 2 (sum of the states in the isolated occupied band region, contains three individual states) (individual charge states shown in Figure S16), 3a (hole trap state, occupied), and 3b (electron trap state, empty). A table of transition energies between the VBM and orbital of interest is seen as an insert within the pDOS. The transition between the VBM and the empty electron trap state (1 → 3b) is 1.2 eV, consistent with the experimentally observed D_3_ defect.

To try and understand the chemical origins of the observed defect DOS, we now turn our attention to DFT calculations. Earlier DFT calculations on the ground state of bulk BA_2_PbI_4_ found several low-formation energy defects in this material.^51^ The results from that study indicated that most common defects were benign and only caused negligible perturbations to the electronic structure and optoelectronic properties, consistent with other 2D perovskite experiments.^52−54^ The lone exception in that case were halogen defects that break the electron spin pairing, such as I vacancies and I interstitials that lead to localized trap states. For this study, to understand the defect physics in the first few nm of the material (i.e., the probing depth in the CFSYS), we created a BA_2_PbI_4_ slab and used DFT to determine if common material defects lead to shallow or deep trap states. Since I vacancies and interstitials were the defects of most interest in the bulk systems, we looked at 4 different types of I vacancies and interstitials, with different locations in the slab, see SI Section S3, Figures S12–S16. These defects have been found to be the most readily created point defects with low formation energies in single-layered BA_2_PbI_4_.^55^ The computational methods are discussed in Section S3 of the SI. We again found that most defects resulted in shallow trap states, and those results are also shown in the SI. We find that all the I interstitials we modeled did in fact create localized midgap trap states. The “Bulk” like I interstitial (I_i_ 4) system along the center of the PbI layer results in the energetically deepest localized trap states, and they are the focus of our deeper analysis, however, we note that the I vacancies found in S15a–c in the SI may also play a role in explaining shallow defects such as D_1_ for example. We also calculated the DOS before and after defect-induced lattice reorganization and confirmed that even then, I vacancies lead to shallow defects, as shown in Figure S17 and Table S1. Figure 10 shows the spin polarized projected density of states for the I interstitial (I_i_ 4) system as well as the energy differences between electronic states as they would map to experimental values in Figure 9. The pDOS shows an isolated occupied band region ca. 0.3 eV above the VBM, containing three individual states (2a, 2b and 2c). They are not resolved in the pDOS in Figure 10, but charge distribution analysis reveals them to be delocalized hole trap states (individual charge states shown in Figure S16). While the three individual states (2a, 2b and 2c) are delocalized parallel to the plane of the slab, they are localized to a single layer and differ by their location on the slab: 2a is on the center layer, 2b and 2c are on the surface layers. In contrast, the charge distribution analysis of trap “3” reveals a highly localized trap state near the I interstitial site. Due to the uneven number of excess electrons of the additional I in the system, state 3a becomes a hole trap state (filled orbital), while 3b becomes an electron trap state (empty orbital), despite having the same highly localized charge distribution. It is the 1 to 3b transition that should be compared to the D_3_ transition in experiment. Overall, a remarkable agreement is found between the experimental results and DFT calculations, as discussed next.

All experimental transition energies correlate well with energetic distances between the valence band edge and trap state energies associated with bulk I interstitials: D_1_ (0.27 eV) corresponds to the calculated transition 1 (VBM) → 2a,b,c (0.26–0.32 eV), D_2_ (0.81 eV) corresponds to transitions 1 (VBM) → 3a (0.79 eV) and most interestingly D_3_ (1.20 eV) corresponds to the deep trap state formation with transition 1 (VBM) → 3b (1.19 eV). Our calculations indicate that surface defects are less detrimental than deep bulk interstitial point defects. We note that although defect-to-defect transitions are also possible, we focused our attention on VBM-to-defect transitions as the more likely ones, since they involve an extended and a localized state–whereas localized-to-localized transitions are very unlikely due to the lack of overlap between the wave functions, and the very low concentration of the initial and final states. We suggest that upon subsequent ionization during CFSYS measurements, Frenkel defects are created in the form of surface I migration into deep I interstitial sites. I migration has been previously studied in 2D perovskites, and a low energy barrier for migration between a single layer was found with less than 0.8 eV, while the migration barrier for I migration between layers is less than 0.5 eV. This could explain why the I_4_ defect shown corresponds most closely to experimental results.^52^ This suggests that surface interstitials and vacancies are more likely to migrate deeper within the 2D perovskite (becoming more “bulk” like defects) than staying within a single layer of the material. This would result in a more substantial growth of the signal originating from I_4_, and hence a much more pronounced increase of the D_3_ feature, as observed experimentally from the CFSYS measurements.

Vacuum-induced degradation of BA_2_PbI_4_ was already reported by Hofstetter et al.^56^ However, the relation between the degradation mechanism and the microscopic processes occurring inside the material was not resolved. The agreement between our experimental and calculation results suggests that the most prominent defect, D_3_, which increases in density upon irradiation with UV light under UHV conditions, is related to the formation of I interstitials, which are found to be the first step in the degradation mechanism.

In contrast to the 2D HaPs, only weak UV-induced degradation was observed for 3D HaPs.^28^ This could be related to inferior self-healing properties of the 2D perovskite surface. Since the probing depth of the CFSYS is between 5–10 nm, our finding suggests that although for the 2D HaP, self-healing of the bulk was found to be more efficient than for the 3D counterparts,^57^ in terms of the surface, self-healing in 2D HaPs is far less efficient than in the 3D HaPs, and UV-induced formation of Iodide interstitials in 2D HaPs is not reversible.

## Conclusions

Defects and self-trapped excitonic states are critical factors limiting charge transport in 2D perovskites, but fundamental knowledge of both processes is limited. The complementary methods of PL, SPV, CLIMAT, and CFSYS demonstrated that spectral features in the range between 2.36 and 2.5 eV are dominated by exciton generation and diffusion whereas transitions below 2.36 eV are caused by defect states and not by self-trapped excitons, and hence can probably be mitigated, depending on the preparation route. Since sub-band gap SPV spectroscopy relies on monochromatic light absorption below the band gap, the observed sub-band gap transitions serve as a direct experimental proof that emission from defect states can explain sub-band gap emission in 2D HaPs, which allows to exclude the Self Trapped Exciton model as an alternative explanation. In addition, ab initio calculations suggest that common defects readily occur and yield electronic states that agree well with what is observed experimentally. We further observe fast diffusion and dissociation of photogenerated excitons in the 2D HaPs, resulting in relatively high charge separation efficiency for a material with a large exciton binding energy. In addition, the UV radiation-induced iodide interstitials formation that results in the creation of midgap states observed in our study suggests that special care needs to be taken upon characterization of 2D HaPs using photon energies in the UV/X-ray range, i.e., He-UPS and/or XPS, widely used in the materials science community.

The unique combination of optical (PL), photoelectrical (SPV), photoemission (CFSYS) and computational techniques used in our study can be applied in the future to a variety of novel hybrid organic–inorganic semiconductors, in order to reliably determine the mechanisms responsible for degradation mechanisms as well as any sub-band gap emission or transport losses. This will help with further development and optimization of optoelectronic devices based on these materials.

## Supporting Information Available

Additional experimental data and calculation results, as well as detailed experimental and calculation procedures (PDF)

## References

[ref1] Ghimire S.; Klinke C. Two-Dimensional Halide Perovskites: Synthesis, Optoelectronic Properties, Stability, and Applications. Nanoscale 2021, 13 (29), 12394–12422. 10.1039/D1NR02769G. 34240087

[ref2] Blancon J. C.; Even J.; Stoumpos C. C.; Kanatzidis M. G.; Mohite A. D. Semiconductor Physics of Organic–Inorganic 2D Halide Perovskites. Nat. Nanotechnol. 2020, 15 (12), 969–985. 10.1038/s41565-020-00811-1. 33277622

[ref3] Xiao Y.; Xue C.; Wang X.; Liu Y.; Yang Z.; Liu S. Bulk Heterostructure BA2PbI4/MAPbI3Perovskites for Suppressed Ion Migration to Achieve Sensitive X-Ray Detection Performance. ACS Appl. Mater. Interfaces 2022, 14 (49), 54867–54875. 10.1021/acsami.2c17715. 36449273

[ref4] Mahmud M. A.; Duong T.; Peng J.; Wu Y.; Shen H.; Walter D.; Nguyen H. T.; Mozaffari N.; Tabi G. D.; Catchpole K. R.; Weber K. J.; White T. P. Origin of Efficiency and Stability Enhancement in High-Performing Mixed Dimensional 2D-3D Perovskite Solar Cells: A Review. Adv. Funct. Mater. 2022, 32 (3), 2009164 10.1002/adfm.202009164.

[ref5] Gharibzadeh S.; Abdollahi Nejand B.; Jakoby M.; Abzieher T.; Hauschild D.; Moghadamzadeh S.; Schwenzer J. A.; Brenner P.; Schmager R.; Haghighirad A. A.; Weinhardt L.; Lemmer U.; Richards B. S.; Howard I. A.; Paetzold U. W. Record Open-Circuit Voltage Wide-Bandgap Perovskite Solar Cells Utilizing 2D/3D Perovskite Heterostructure. Adv. Energy Mater. 2019, 9 (21), 1803699 10.1002/aenm.201803699.

[ref6] Li J.; Wang J.; Ma J.; Shen H.; Li L.; Duan X.; Li D. Self-Trapped State Enabled Filterless Narrowband Photodetections in 2D Layered Perovskite Single Crystals. Nat. Commun. 2019, 10 (1), 806 10.1038/s41467-019-08768-z. 30778073 PMC6379360

[ref7] Li J.; Wang H.; Li D. Self-Trapped Excitons in Two-Dimensional Perovskites. Front. Optoelectron. 2020, 13 (3), 225–234. 10.1007/s12200-020-1051-x. 36641579 PMC9743880

[ref8] Williams R. T.; Song K. S. The Self-Trapped Exciton. J. Phys. Chem. Solids 1990, 51 (7), 679–716. 10.1016/0022-3697(90)90144-5.

[ref9] Kitaura M.; Nakagawa H. Self-Trapped Exciton and Recombination Luminescence in PbCl2, PbBr2 and Their Mixed Crystals. J. Lumin. 1997, 72–74, 883–884. 10.1016/S0022-2313(96)00273-6.

[ref10] Lushchik A.; Kirm M.; Lushchik C.; Martinson I.; Zimmerer G. Luminescence of Free and Self-Trapped Excitons in Wide-Gap Oxides. J. Lumin. 2000, 87–89, 232–234. 10.1016/S0022-2313(99)00271-9.

[ref11] Ismail-Beigi S.; Louie S. G. Self-Trapped Excitons in Silicon Dioxide: Mechanism and Properties. Phys. Rev. Lett. 2005, 95 (15), 156401 10.1103/PhysRevLett.95.156401. 16241743

[ref12] Sildos I.; Suisalu A.; Aarik J.; Sekiya T.; Kurita S. Self-Trapped Exciton Emission in Crystalline Anatase. J. Lumin. 2000, 87–89, 290–292. 10.1016/S0022-2313(99)00318-X.

[ref13] Han Y.; Cheng X.; Cui B.-B. Factors Influencing Self-Trapped Exciton Emission of Low-Dimensional Metal Halides. Mater. Adv. 2023, 4 (2), 355–373. 10.1039/D2MA00676F.

[ref14] Wang Y.; He C.; Tan Q.; Tang Z.; Huang L.; Liu L.; Yin J.; Jiang Y.; Wang X.; Pan A. Exciton-Phonon Coupling in Two-Dimensional Layered (BA)2PbI4 Perovskite Microplates. RSC Adv. 2023, 13 (9), 5893–5899. 10.1039/D2RA06401D. 36816078 PMC9936372

[ref15] Li G.; Zhang Y.; Wang W.; Gao L.; Ren Y.; Che J.; Mo C.; Liu H.; Zhao W.; Lu J.; Ni Z. Correlated Dynamics of Free and Self-Trapped Excitons and Broadband Photodetection in BEA2PbBr4 Layered Crystals. Adv. Opt. Mater. 2022, 10 (12), 2200223 10.1002/adom.202200223.

[ref16] Kahmann S.; Meggiolaro D.; Gregori L.; Tekelenburg E. K.; Pitaro M.; Stranks S. D.; De Angelis F.; Loi M. A. The Origin of Broad Emission in ⟨100≫Two-Dimensional Perovskites: Extrinsic vs Intrinsic Processes. ACS Energy Lett. 2022, 7 (12), 4232–4241. 10.1021/acsenergylett.2c02123. 36531144 PMC9745793

[ref17] Fang H. H.; Tekelenburg E. K.; Xue H.; Kahmann S.; Chen L.; Adjokatse S.; Brocks G.; Tao S.; Loi M. A. Unraveling the Broadband Emission in Mixed Tin-Lead Layered Perovskites. Adv. Opt. Mater. 2022, 11, 2202038 10.1002/adom.202202038.

[ref18] Liang G. Q.; Zhang J. A Machine Learning Model for Screening Thermodynamic Stable Lead-Free Halide Double Perovskites. Comput. Mater. Sci. 2022, 204, 111172 10.1016/j.commatsci.2021.111172.

[ref19] Kahmann S.; Tekelenburg E. K.; Duim H.; Kamminga M. E.; Loi M. A. Extrinsic Nature of the Broad Photoluminescence in Lead Iodide-Based Ruddlesden–Popper Perovskites. Nat. Commun. 2020, 11 (1), 2344 10.1038/s41467-020-15970-x. 32393785 PMC7214453

[ref20] Yin J.; Naphade R.; Gutiérrez Arzaluz L.; Brédas J. L.; Bakr O. M.; Mohammed O. F. Modulation of Broadband Emissions in Two-Dimensional â ¨100â ©-Oriented Ruddlesden-Popper Hybrid Perovskites. ACS Energy Lett. 2020, 5 (7), 2149–2155. 10.1021/acsenergylett.0c01047.

[ref21] Gao H.; Meng C.; Liu B.; Ma X.; Ye H. Conditions of Photo-Induced Defect Generation and Their Luminescence in Two-Dimensional Lead Bromide Perovskites. ACS Appl. Nano Mater. 2023, 6 (23), 21514–21520. 10.1021/acsanm.3c02812.

[ref22] Paritmongkol W.; Powers E. R.; Dahod N. S.; Tisdale W. A. Two Origins of Broadband Emission in Multilayered 2D Lead Iodide Perovskites. J Phys Chem Lett. 2020, 11 (20), 8565–8572. 10.1021/acs.jpclett.0c02214. 32975424

[ref23] Hu H.; Liu Y.; Xie Z.; Xiao Z.; Niu G.; Tang J. Observation of Defect Luminescence in 2D Dion–Jacobson Perovskites. Adv. Opt. Mater. 2021, 9 (24), 1–8. 10.1002/adom.202101423.

[ref24] Guzelturk B.; Winkler T.; Van de Goor T. W. J.; Smith M. D.; Bourelle S. A.; Feldmann S.; Trigo M.; Teitelbaum S. W.; Steinrück H.-G.; de la Pena G. A.; Alonso-Mori R.; Zhu D.; Sato T.; Karunadasa H. I.; Toney M. F.; Deschler F.; Lindenberg A. M. Visualization of Dynamic Polaronic Strain Fields in Hybrid Lead Halide Perovskites. Nat. Mater. 2021, 20 (5), 618–623. 10.1038/s41563-020-00865-5. 33398119

[ref25] Musiienko A.; Ceratti D. R.; Pipek J.; Brynza M.; Elhadidy H.; Belas E.; Betušiak M.; Delport G.; Praus P. Defects in Hybrid Perovskites: The Secret of Efficient Charge Transport. Adv. Funct. Mater. 2021, 31 (48), 2104467 10.1002/adfm.202104467.

[ref26] Levine I.; Vera O. G.; Kulbak M.; Ceratti D.-R.; Rehermann C.; Márquez J. A.; Levcenko S.; Unold T.; Hodes G.; Balberg I.; Cahen D.; Dittrich T. Deep Defect States in Wide-Band-Gap ABX _3_ Halide Perovskites. ACS Energy Lett. 2019, 4 (5), 1150–1157. 10.1021/acsenergylett.9b00709.

[ref27] Das D. K.; Bakthavatsalam R.; Hathwar V. R.; Pallepogu R.; Kundu J. Intrinsic vs. Extrinsic STE Emission Enhancement in Ns2 Ion Doped Metal (Cd, In) Halide Hybrids. J. Mater. Chem. C 2023, 11 (11), 3855–3864. 10.1039/D2TC04361K.

[ref28] Menzel D.; Tejada A.; Al-Ashouri A.; Levine I.; Guerra J. A.; Rech B.; Albrecht S.; Korte L. Revisiting the Determination of the Valence Band Maximum and Defect Formation in Halide Perovskites for Solar Cells: Insights from Highly Sensitive Near–UV Photoemission Spectroscopy. ACS Appl. Mater. Interfaces 2021, 13 (36), 43540–43553. 10.1021/acsami.1c10171. 34472345

[ref29] Dittrich T.; Fengler S. Surface Photovoltage Analysis of Photoactive Materials; World Scientific Europe, 2020.

[ref30] Musiienko A.; Moravec P.; Grill R.; Praus P.; Vasylchenko I.; Pekarek J.; Tisdale J.; Ridzonova K.; Belas E.; Landová L.; Hu B.; Lukosi E.; Ahmadi M. Deep Levels, Charge Transport and Mixed Conductivity in Organometallic Halide Perovskites. Energy Environ. Sci. 2019, 12 (4), 1413–1425. 10.1039/C9EE00311H.

[ref31] Levine I.; Vera O. G.; Kulbak M.; Ceratti D. R.; Rehermann C.; Márquez J. A.; Levcenko S.; Unold T.; Hodes G.; Balberg I.; Cahen D.; Dittrich T. Deep Defect States in Wide-Band-Gap ABX3 Halide Perovskites. ACS Energy Lett. 2019, 4 (5), 1150–1157. 10.1021/acsenergylett.9b00709.

[ref32] Levine I.; Shimizu K.; Lomuscio A.; Kulbak M.; Rehermann C.; Zohar A.; Abdi-Jalebi M.; Zhao B.; Siebentritt S.; Zu F.; Koch N.; Kahn A.; Hodes G.; Friend R. H.; Ishii H.; Cahen D. Direct Probing of Gap States and Their Passivation in Halide Perovskites by High-Sensitivity, Variable Energy Ultraviolet Photoelectron Spectroscopy. J. Phys. Chem. C 2021, 125 (9), 5217–5225. 10.1021/acs.jpcc.0c11627.

[ref33] Dittrich T.; Fengler S.; Franke M. Transient Surface Photovoltage Measurement over 12 Orders of Magnitude in Time. Rev. Sci. Instrum. 2017, 88 (5), 053904 10.1063/1.4983079. 28571417

[ref34] Stoumpos C. C.; Cao D. H.; Clark D. J.; Young J.; Rondinelli J. M.; Jang J. I.; Hupp J. T.; Kanatzidis M. G. Ruddlesden-Popper Hybrid Lead Iodide Perovskite 2D Homologous Semiconductors. Chem. Mater. 2016, 28 (8), 2852–2867. 10.1021/acs.chemmater.6b00847.

[ref35] Sheikh T.; Shinde A.; Mahamuni S.; Nag A. Possible Dual Bandgap in (C4H9NH3)2PbI4 2D Layered Perovskite: Single-Crystal and Exfoliated Few-Layer. ACS Energy Lett. 2018, 3 (12), 2940–2946. 10.1021/acsenergylett.8b01799.

[ref36] Sheikh T.; Nawale V.; Pathoor N.; Phadnis C.; Chowdhury A.; Nag A. Molecular Intercalation and Electronic Two Dimensionality in Layered Hybrid Perovskites. Angew. Chem., Int. Ed. 2020, 59 (28), 11653–11659. 10.1002/anie.202003509. 32243656

[ref37] Kronik L.; Shapira Y. Surface Photovoltage Spectroscopy of Semiconductor Structures: At the Crossroads of Physics, Chemistry and Electrical Engineering. Surf. Interface Anal. 2001, 31 (10), 954–965. 10.1002/sia.1132.

[ref38] Silver S.; Yin J.; Li H.; Brédas J. L.; Kahn A. Characterization of the Valence and Conduction Band Levels of n = 1 2D Perovskites: A Combined Experimental and Theoretical Investigation. Adv. Energy Mater. 2018, 8 (16), 1–7. 10.1002/aenm.201703468.

[ref39] Ziegler J. D.; Lin K. Q.; Meisinger B.; Zhu X.; Kober-Czerny M.; Nayak P. K.; Vona C.; Taniguchi T.; Watanabe K.; Draxl C.; Snaith H. J.; Lupton J. M.; Egger D. A.; Chernikov A. Excitons at the Phase Transition of 2D Hybrid Perovskites. ACS Photonics 2022, 9 (11), 3609–3616. 10.1021/acsphotonics.2c01035.

[ref40] Blancon J. C.; Tsai H.; Nie W.; Stoumpos C. C.; Pedesseau L.; Katan C.; Kepenekian M.; Soe C. M. M.; Appavoo K.; Sfeir M. Y.; Tretiak S.; Ajayan P. M.; Kanatzidis M. G.; Even J.; Crochet J. J.; Mohite A. D. Extremely Efficient Internal Exciton Dissociation through Edge States in Layered 2D Perovskites. Science 2017, 355 (6331), 1288–1292. 10.1126/science.aal4211. 28280250

[ref41] Simbula A.; Wu L.; Pitzalis F.; Pau R.; Lai S.; Liu F.; Matta S.; Marongiu D.; Quochi F.; Saba M.; Mura A.; Bongiovanni G. Exciton Dissociation in 2D Layered Metal-Halide Perovskites. Nat. Commun. 2023, 14 (1), 4125 10.1038/s41467-023-39831-5. 37433858 PMC10336065

[ref42] Musiienko A.; Yang F.; Gries T. W.; Frasca C.; Friedrich D.; Al-Ashouri A.; Sağlamkaya E.; Lang F.; Kojda D.; Huang Y. T.; Stacchini V.; Hoye R. L. Z.; Ahmadi M.; Kanak A.; Abate A. Resolving Electron and Hole Transport Properties in Semiconductor Materials by Constant Light-Induced Magneto Transport. Nat. Commun. 2024, 15 (1), 316 10.1038/s41467-023-44418-1. 38182589 PMC10770130

[ref43] Milot R. L.; Sutton R. J.; Eperon G. E.; Haghighirad A. A.; Martinez Hardigree J.; Miranda L.; Snaith H. J.; Johnston M. B.; Herz L. M. Charge-Carrier Dynamics in 2D Hybrid Metal-Halide Perovskites. Nano Lett. 2016, 16 (11), 7001–7007. 10.1021/acs.nanolett.6b03114. 27689536

[ref44] Motti S. G.; Kober-Czerny M.; Righetto M.; Holzhey P.; Smith J.; Kraus H.; Snaith H. J.; Johnston M. B.; Herz L. M. Exciton Formation Dynamics and Band-Like Free Charge-Carrier Transport in 2D Metal Halide Perovskite Semiconductors. Adv. Funct. Mater. 2023, 33 (32), 2300363 10.1002/ADFM.202300363.

[ref45] Gélvez-Rueda M. C.; Hutter E. M.; Cao D. H.; Renaud N.; Stoumpos C. C.; Hupp J. T.; Savenije T. J.; Kanatzidis M. G.; Grozema F. C. Interconversion between Free Charges and Bound Excitons in 2D Hybrid Lead Halide Perovskites. J. Phys. Chem. C 2017, 121 (47), 26566–26574. 10.1021/acs.jpcc.7b10705. PMC5712865 29218073

[ref46] Ludwig G. W.; Watters R. L. Drift and Conductivity Mobility in Silicon. Phys. Rev. 1956, 101 (6), 1699 10.1103/PhysRev.101.1699.

[ref47] Silver M.; Winborne G.; Adler D.; Cannella V. Electron Mobility in Hydrogenated Amorphous Silicon under Single and Double Injection. Appl. Phys. Lett. 1987, 50 (15), 983–985. 10.1063/1.98005.

[ref49] Zhang F.; Ullrich F.; Silver S.; Kerner R. A.; Rand B. P.; Kahn A. Complexities of Contact Potential Difference Measurements on Metal Halide Perovskite Surfaces. J. Phys. Chem. Lett. 2019, 10 (4), 890–896. 10.1021/acs.jpclett.8b03878. 30739454

[ref50] Zhang F.; Silver S. H.; Noel N. K.; Ullrich F.; Rand B. P.; Kahn A. Ultraviolet Photoemission Spectroscopy and Kelvin Probe Measurements on Metal Halide Perovskites: Advantages and Pitfalls. Adv. Energy Mater. 2020, 10 (26), 1–7. 10.1002/aenm.201903252.

[ref51] Perez C. M.; Ghosh D.; Prezhdo O.; Nie W.; Tretiak S.; Neukirch A. Point Defects in Two-Dimensional Ruddlesden-Popper Perovskites Explored with Ab Initio Calculations. J. Phys. Chem. Lett. 2022, 13, 5213–5219. 10.1021/acs.jpclett.2c00575. 35670577

[ref52] Imani R.; Ghasempour Ardakani A.; Moradi M.; Jacobsson T. J.; Pazoki M. Modelling Iodine Diffusion in 2D-Perovskites as a Function of the Length of the Organic Spacer Molecules. Sol. Energy 2024, 272, 112458 10.1016/j.solener.2024.112458.

[ref53] Xiao Z.; Meng W.; Wang J.; Yan Y. Defect Properties of the Two-Dimensional (CH3NH3)2Pb(SCN)2I2 Perovskite: A Density-Functional Theory Study. Phys. Chem. Chem. Phys. 2016, 18 (37), 25786–25790. 10.1039/C6CP05302E. 27604477

[ref54] Song J.; Qian J.; Liu L.; Huang D.; Li Z.; Xu B.; Tian W. Theoretical Study on Defect Properties of Two-Dimensional Multilayer Ruddlesden-Popper Lead Iodine Perovskite. Comput. Mater. Sci. 2021, 194, 110457 10.1016/j.commatsci.2021.110457.

[ref55] Xue H.; Chen Z.; Tao S.; Brocks G. Defects in Halide Perovskites: Does It Help to Switch from 3D to 2D?. ACS Energy Lett. 2024, 9, 2343–2350. 10.1021/acsenergylett.4c00702. 38751970 PMC11091873

[ref56] Hofstetter Y. J.; García-Benito I.; Paulus F.; Orlandi S.; Grancini G.; Vaynzof Y. Vacuum-Induced Degradation of 2D Perovskites. Front. Chem. 2020, 8, 66 10.3389/fchem.2020.00066. 32117889 PMC7031494

[ref57] Aharon S.; Ceratti D. R.; Jasti N. P.; Cremonesi L.; Feldman Y.; Potenza M. A. C.; Hodes G.; Cahen D. 2D Pb-Halide Perovskites Can Self-Heal Photodamage Better than 3D Ones. Adv. Funct. Mater. 2022, 32 (24), 2113354 10.1002/adfm.202113354.

