# Supplementary material for: Revisiting Sub-Band Gap Emission Mechanism in 2D Halide Perovskites: The Role of Defect States
Source: J Am Chem Soc. 2024 Aug 8;146(33):23437–48. doi: 10.1021/jacs.4c06621 (PMC11345761; doi:10.1021/jacs.4c06621)
Supplement: Supplementary file 1 — ja4c06621_si_001.pdf [file ja4c06621_si_001.pdf]

# *Supplementary information for:*

# Revisiting Sub-Bandgap Emission Mechanism in 2D Halide Perovskites: The Role of Defect States

Igal Levine*[1,2], Dorothee Menzel[1], Artem Musiienko[1], Rowan MacQueen[1], Natalia Romano[1], Manuel Vasquez-Montoya[1], Eva Unger[1], Carlos Mora Perez[3,4], Aaron Forde[3], Amanda J. Neukirch[3], Lars Korte[1] and Thomas Dittrich[1]

[1]Helmholtz-Zentrum Berlin für Materialien und Energie GmbH, Division Solar Energy, Kekuléstraße 5, 12489 Berlin, Germany

[2]Institute of Chemistry and The Center for Nanoscience and Nanotechnology, The Hebrew University, Jerusalem 91904, Israel

[3]Theoretical Physics and chemistry of Materials, Los Alamos National Laboratory, Los Alamos, New Mexico 87545, United States

[4]Department of Chemistry, University of Southern California, Los Angeles, California 90089, United States

*****Email: igal.levine@mail.huji.ac.il

## *S1 – Additional data and notes*

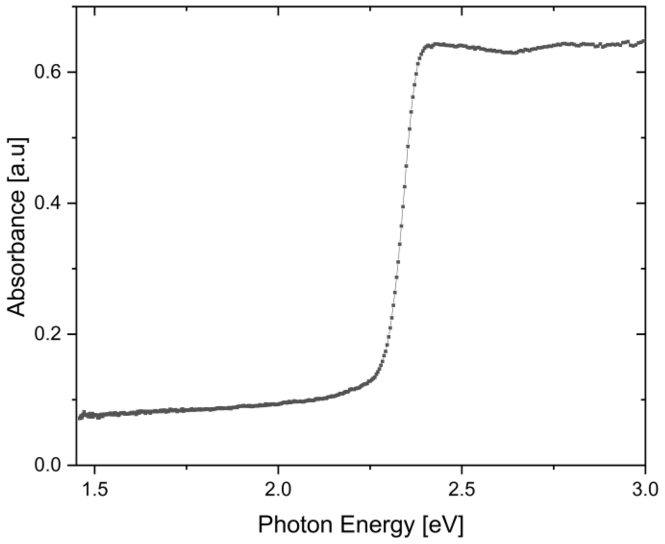


Figure S1 :UV-Vis absorption spectrum of a flake of a $BA_2PbI_4$ single crystal

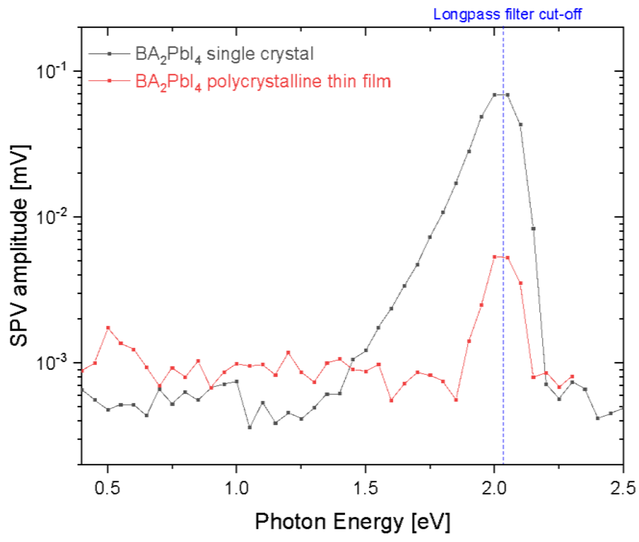


Figure S2: SPV amplitude spectra (combined -x and -y components) of a freshly peeled $BA_2PbI_4$ single crystal (black) and polycrystalline film (red).

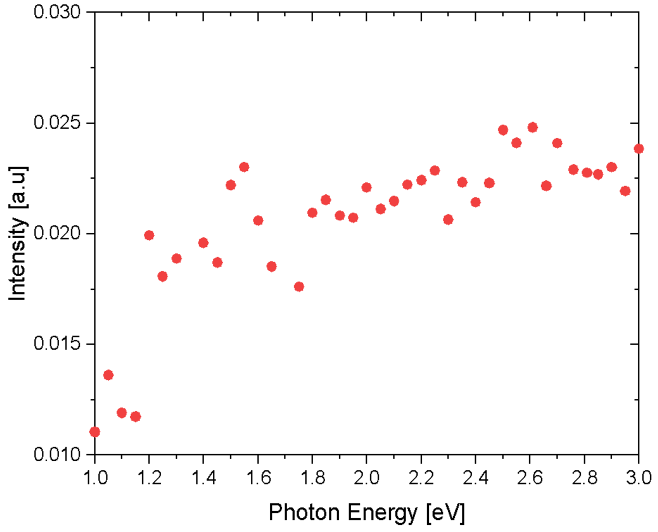


Figure S3: Spectrum of the photon flux of the laser pulses.

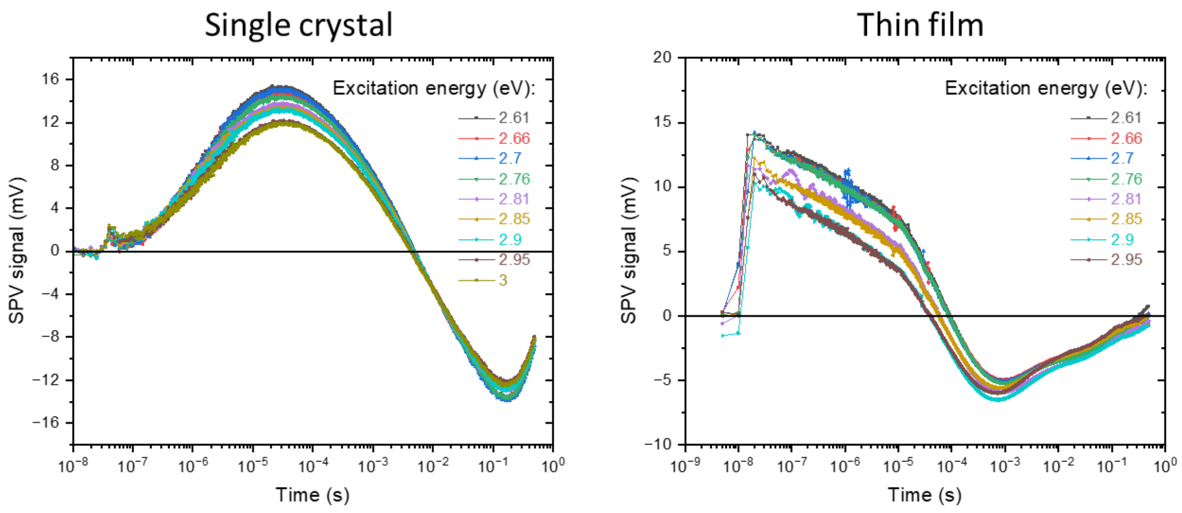


Figure S4: Tr-SPV decays of the BA2PbI4 single crystal (left) and polycrystalline thin film deposited on ITO (right), for excitations above the bandgap ($E_{ex}$> 2.6 eV)

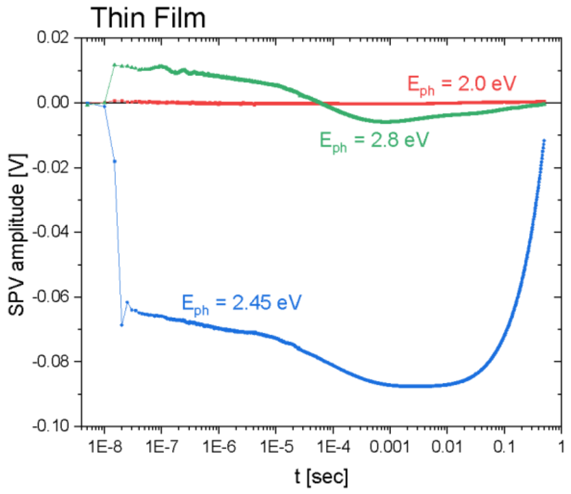


Figure S5 - Selected SPV transients at different laser excitation energies for a $BA_2PbI_4$ polycrystalline thin film deposited on ITO: 2.0 eV (red), 2.45 eV (blue) and 2.8 eV (green)

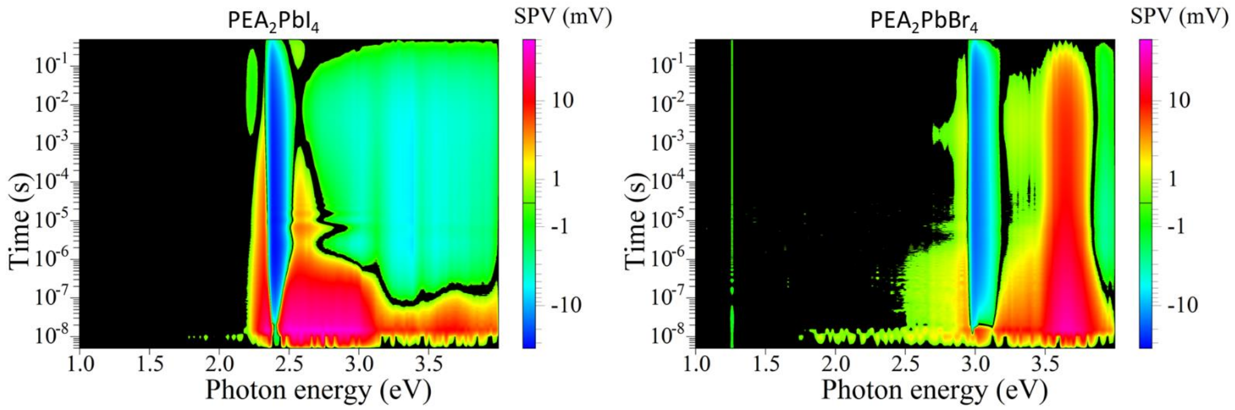

Figure S6 – transient SPV measurements of $PEA_2PbI_4$ and $PEA_2PbBr_4$ polycrystalline thin films deposited on ITO.

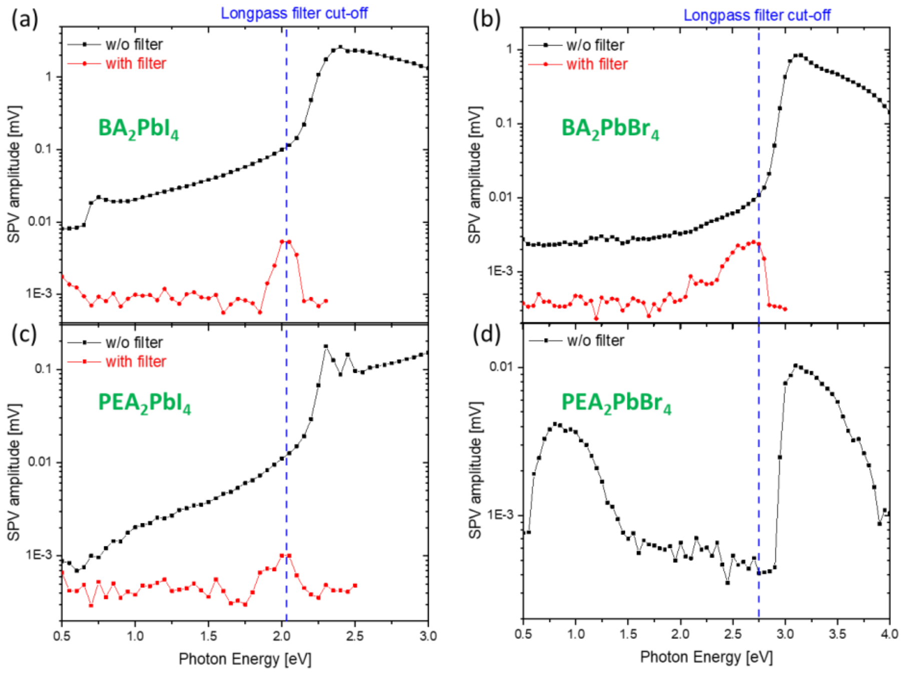


Figure S7: SPV amplitude spectra (combined -x and -y components) of (a) $BA_2PbI_4$, (b) $BA_2PbBr_4$, (c) $PEA_2PbI_4$ and (d) $PEA_2PbBr_4$ polycrystalline thin films deposited on ITO. For $PEA_2PbBr_4$, since the SPV signals were low even without the longpass filter, no stray light effects were need to be suppressed and hence no longpass filter was required

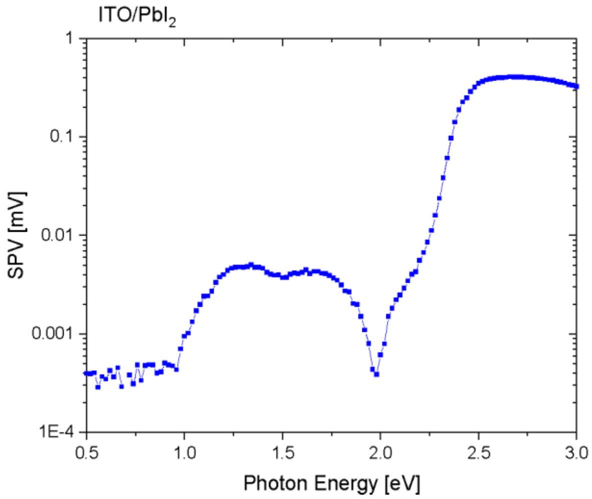


Figure S8: SPV amplitude spectra (combined -x and -y components) of $PbI_2$ thin film spin coated on ITO from a 1M DMSO solution

## ***S2 – CFSYS modelling***

The raw data as a function of the excitation photon energy / binding energy, for both the single crystal and the polycrystalline thin film are shown in Fig. S9:

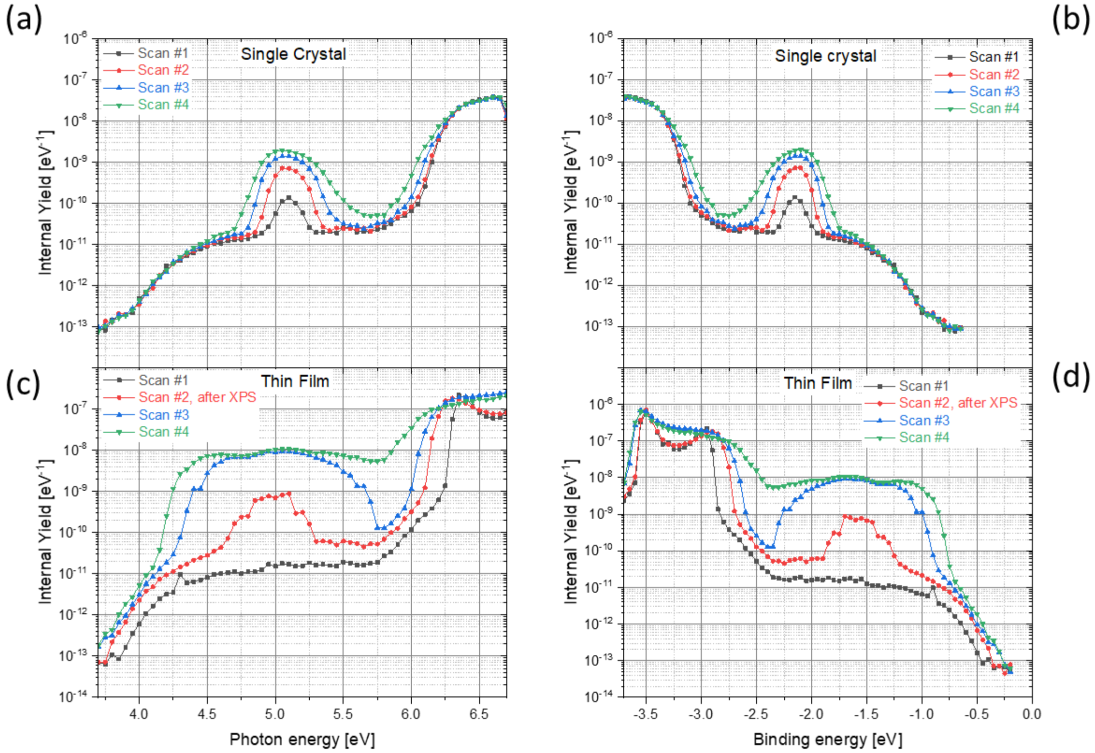


Figure S9: CFSYS measurement results as a function of the excitation photon energy (left) and binding energy (right) for the BA2PbI4 single crystal (top), and of a freshly prepared polycrystalline thin film (bottom)

As can be seen from Figure S9b&d, substantial charging was observed for the single crystal (~ 1 eV) as well as for the thin film (~0.5 eV). Negative charging towards higher binding energies could be a result of the poor conductivity of $BA_2PbI_4$. In order to convert the x-axis to the energetic distance from the VBM ($E-E_{VBM}$), the VBM position was determined by fitting the experimental data using a model, based on a polylogarithmic function, which combines a parabolic band edge and an exponential band tail in one single equation.[1] The resulting VBM position was determined to be at a binding energy of 3.34 eV. The different defects ($D_1$-$D_4$) were fitted as gaussian peaks, and the fitting results are shown in Figure S10. The applied constraints on the defect position is shown in Figure S11a, the obtained defect amplitude in Figure S11b, and the tail parameter, which is essentially the inverse of the slope of the exponential band tail in Figure S11c.

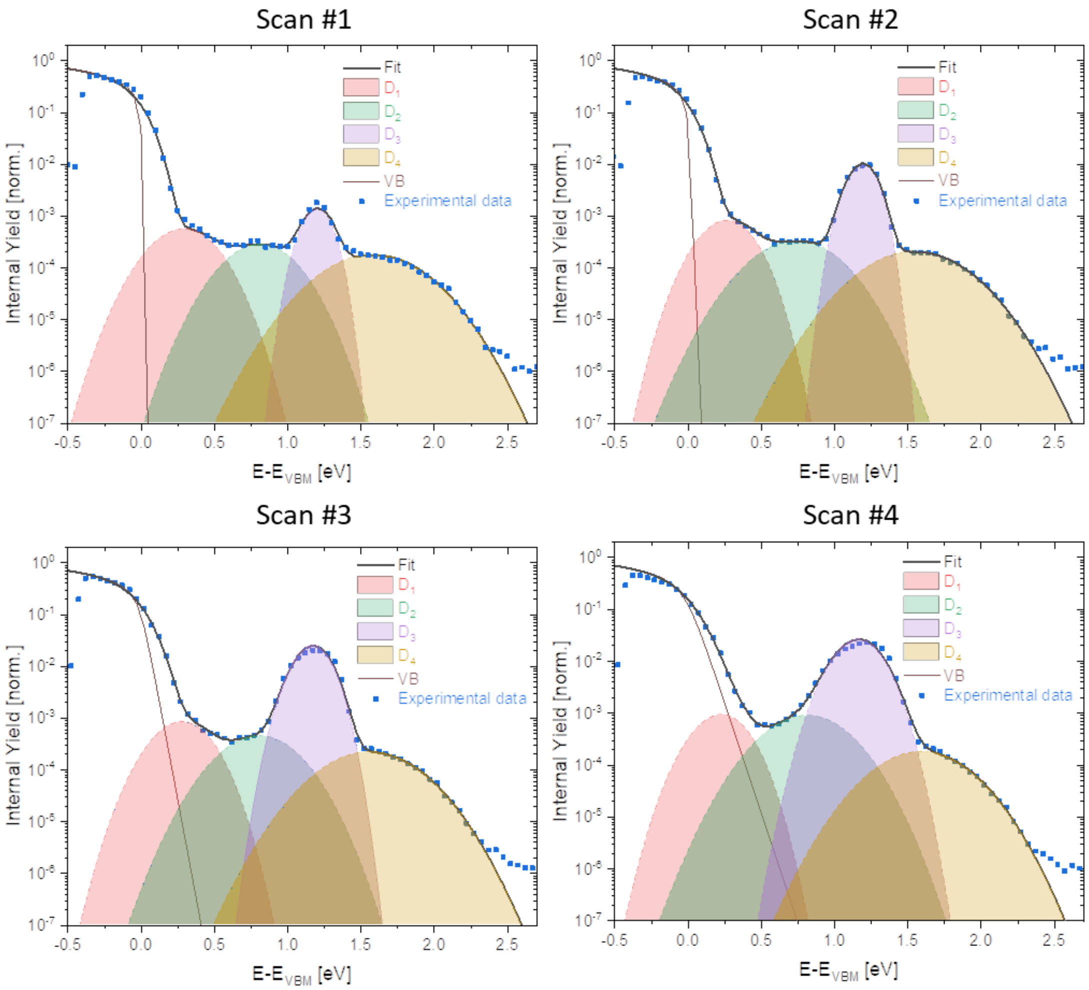


Figure S10: Deconvolution of the CFSYS measurements (4 subsequent scans) of a $BA_2PbI_4$ single crystal.

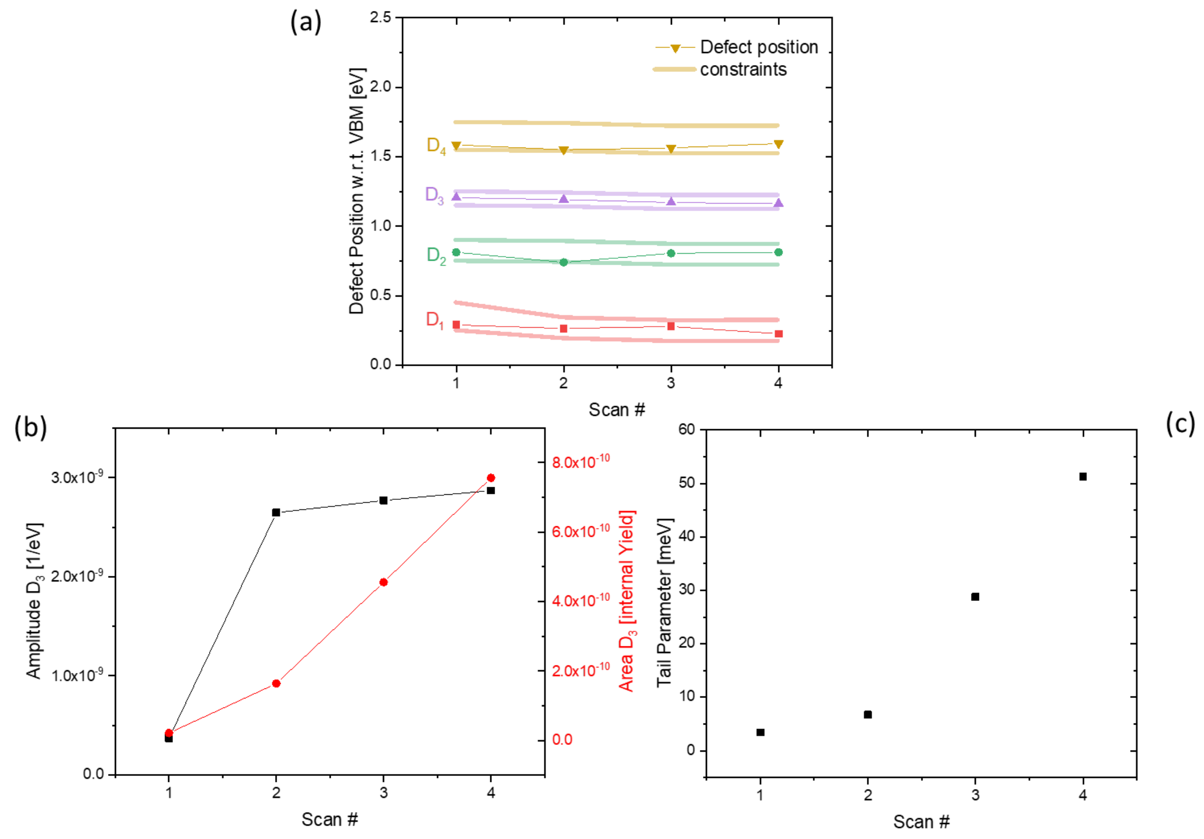


Figure S11: (a) The obtained defect positions as a function of scan number, including the fitting constraints used; (b) The change in the amplitude (black) and area (red) of defect $D_3$ as a function of the scan number; and (c) The extracted tail parameter as a function of scan number.

## ***S3-Theoretical procedures***

The pristine 2D-RP perovskite $BA_2PbI_4$ (BA=butylammonium) for the n=1 layer, perovskite composition, is seen in Figure S10. A previous report has shown that a 2×1×2 model size for the 2D-bulk system is a sufficiently large model which captures defects' effects on the electronic structure.[2] The supercell was designed to minimize defect-defect interactions within the layers while maximizing computational efficiency. Thus, we utilized models built as a 2×2×2 supercell (8-unit primitive cells) with BA-termination on exposed surfaces and a 20 Å vacuum between layers. All investigated defects are visualized with their relative layer location in Figure S13 (Iodine vacancy models) and Figure S14 (Iodine interstitial models). All calculations were performed at the Density Functional Theory (DFT)[3,4] level with the projector-augmented-wave (PAW) potentials[5,6] and a generalized gradient approximation (GGA)[7] functional, PBE.[8] All calculations were done within the Vienna Ab initio Simulation Package (VASP).[9–12] The POTCAR versions included the following PAW PBE per species: PAW_PBE C 08Apr2002, PAW_PBE N 08Apr2002, PAW_PBE H 15Jun2001, PAW_PBE Pb_d 06Sep2000, PAW_PBE I 08Apr2002. Given the BA ligand's flexibility, the Hellmann-Feynman forces' structures were set to lower than 0.03 eV/ Å during the geometry optimization. We utilized a high ENCUT value of 520 eV for all calculations (spin-unpolarized and spin-polarized). To accurately probe the electronic structure of the slab system, we set the KPOINTS along the high-symmetry k-path of Γ-X-U-Z-Γ. Our previous results on bulk system[2]

and Figure S12c show that the bandgap is at the gamma point (Γ → Γ). Therefore, all defect systems were only evaluated at the gamma point for efficiency. We have previously shown that PBE calculations are sufficient for capturing the formation of trap states.[2] Thus, a high theory-level calculation, such as spin-orbit coupling (SOC), was omitted from this study.

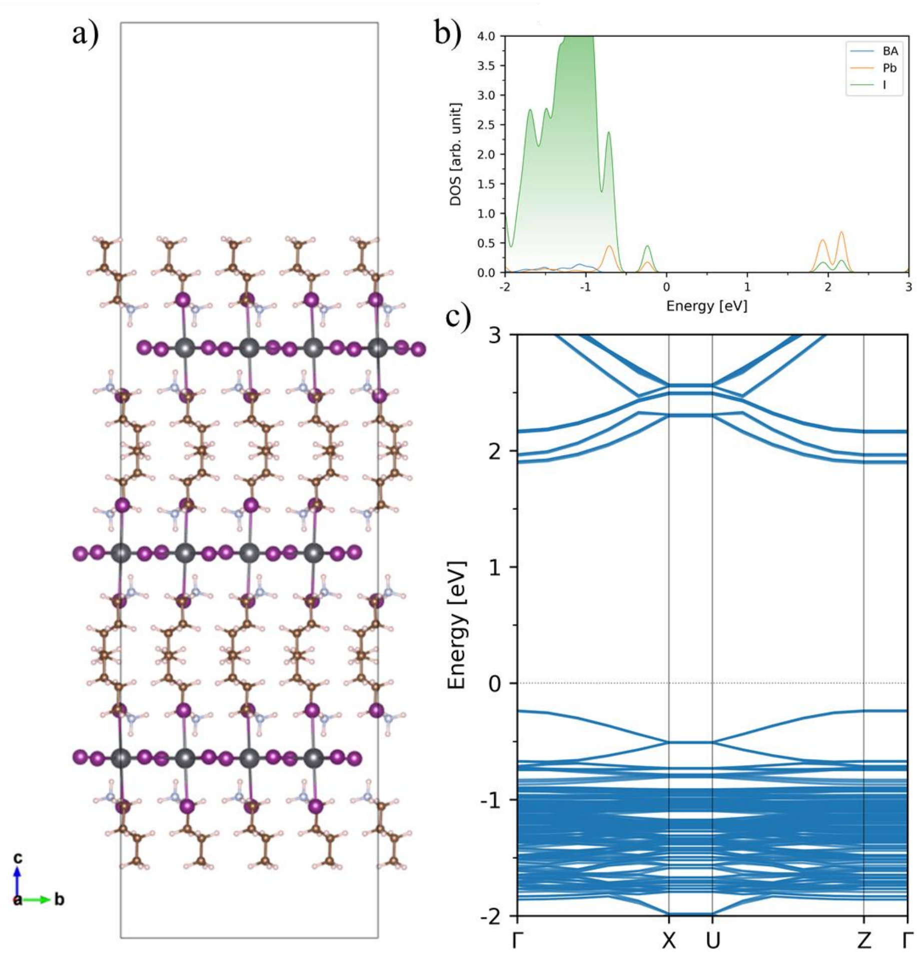


Figure S12: a) The pristine 2D-RP perovskite $BA_2MA_{n-1}PbnI_{3n+1}$ (BA=butylammonium) for the n=1 layer, 2×2×2 supercell (8-unit primitive cells) with BA-termination on exposed surfaces and a 20 Å vacuum. b) The pristine system's projected density of states (pDOS): BA ligand (blue), Pb (orange), I (green). The Fermi energy ($E_F$) is set to zero value. c) The band structure of the pristine system along the high-symmetry path (Γ-X-U-Z-Γ).

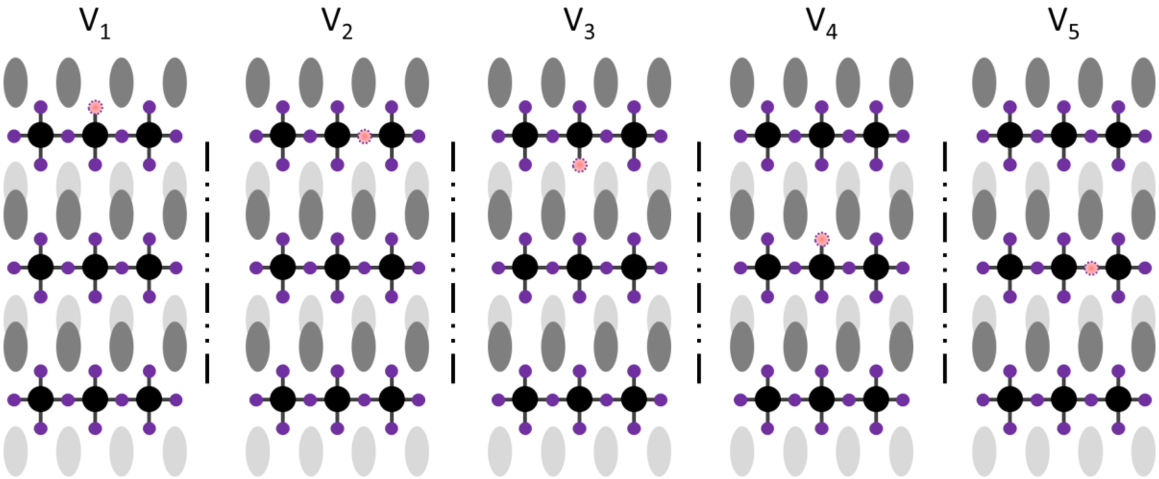


Figure S13: Cartoon representation of iodine vacancy systems.

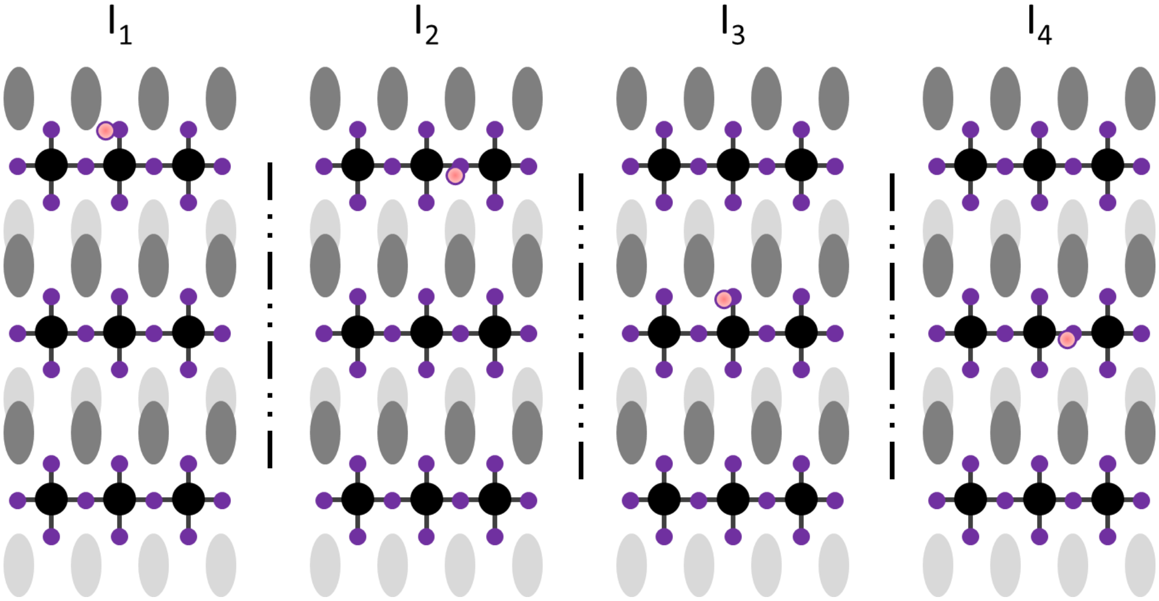


Figure S14: Cartoon representation of Iodine interstitial systems.

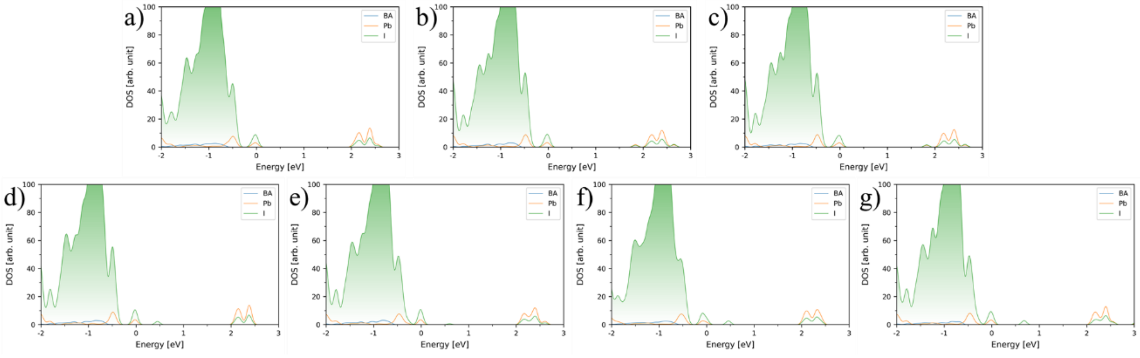


Figure S15**:** The projected density of states (pDOS) of select systems with spin-nonpolarized calculation for initial screening: BA ligand (blue), Pb (orange), I (green). a)$V_I$:1, b) $V_I$:2, c) $V_I$:5, d) $I_I$:1 ,e) $I_I$:2, f) $I_I$:3, g) $I_I$:4

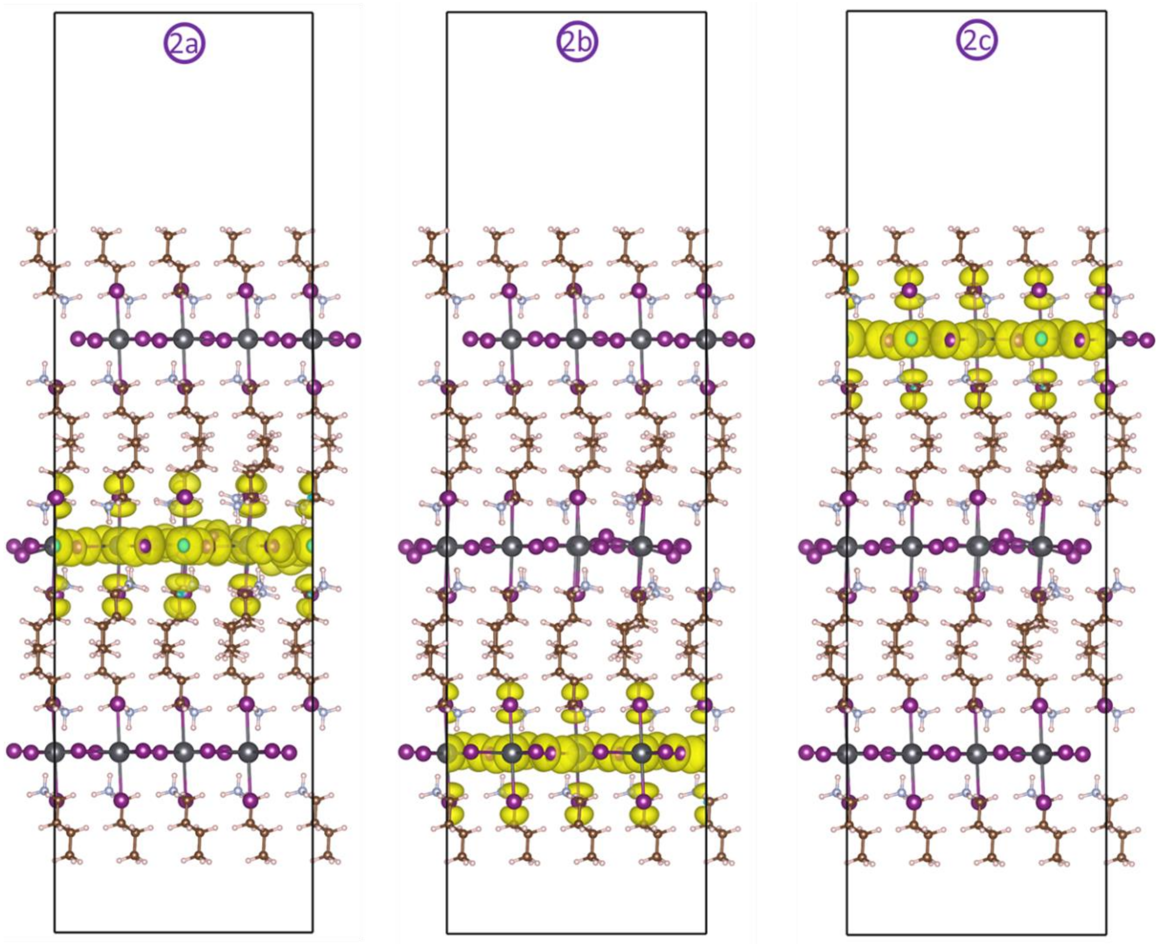


Figure S16. Charge density of the isolated occupied band region of three individual states.

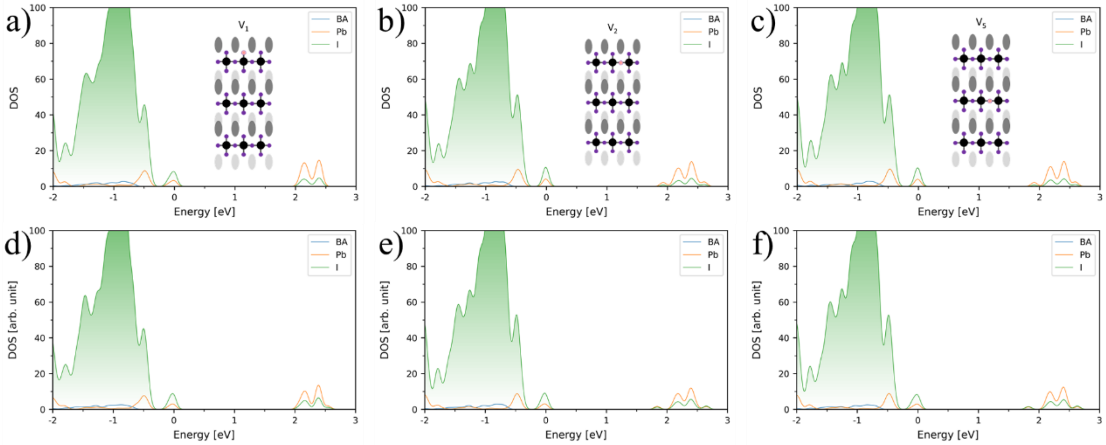


Figure S17**:** The projected density of states (pDOS) before (a,b,c) and after (d,e,f) energy optimization for $V_I$:1, $V_I$:2, and $V_I$:5, respectively. (BA ligand (blue), Pb (orange), I (green))

| Vacancy | 1 | | | 2 | | | 5 | | |
|---|---|---|---|---|---|---|---|---|---|
| | Unoptimized | Optimized | **Difference** | Unoptimized | Optimized | **Difference** | Unoptimized | Optimized | **Difference** |
| CBM | 2.104 | 2.126 | **0.022** | 2.128 | 2.124 | **-0.004** | 2.140 | 2.139 | **-0.001** |
| Trap | 2.079 | 2.068 | **-0.012** | 1.930 | 1.834 | **-0.096** | 1.913 | 1.824 | **-0.089** |
| VBM | 0.000 | 0.000 | **0.000** | 0.000 | 0.000 | **0.000** | 0.000 | 0.000 | **0.000** |

Table S1**:** The differences in the calculated energetic positions before and after energy relaxation for several Iodide vacancies. All values in units of eV.

## *S4 – Experimental procedures*

### *$BA_2PbI_4$ single crystal preparation and structural characterization*

Butylammonium, $C_4H_9NH_3$ (BA) lead iodide ($BA_2PbI_4$) was crystallized using the slow-cooling method with minor modifications.[13] 5.045 mmol (1.126 g) PbO (ACS reagent, ≥99.0%, Sigma-Aldrich) was dissolved in 5 mL HI (57% in $H_2O$, Sigma-Aldrich) and 850 $\mu$L hypophosphorous acid solution (50 wt. % in $H_2O$, Sigma-Aldrich) in an 18 mL vial. After tightly screwing the vial's cap, the mixture was stirred (magnetic stirrer) and heated on a hot plate that was set to 110 ℃. The color of the mixture changed from black to clear yellow within a minute. The stirring and heating continued until full dissolution of the PbO (1-2 hours). In the meantime, in an ice-bath, 3 mL of cooled HI (at ~4 ℃) were mixed with 494 $\mu$L butylamine (99.5%, Sigma-Aldrich) by vigorously stirring the HI with a magnetic stirrer and adding the butylamine dropwise. The vial was then tightly sealed and stirring continued until no vapor was seen in the upper part of the vial. Once the two above mixtures were ready, the HI+butylamine mixture was added, dropwise, to the Pb-containing vial while continuing to stir and heat. This led to the formation of an orange powder. Then the vial was tightly sealed and heated, while stirring, until the orange powder fully dissolved (the hotplate was set for this purpose to 140-170 ℃ for 10-20 minutes). Once the solution was perfectly clear, we carefully took the magnet out of the vial, tightly sealed it again, and transferred it into a system for controlled slow cooling that contained a silicone-oil bath, which was preheated to 105 ℃. The silicone oil bath sat in a closed (Pyrex) glass container to maintain a uniform temperature. The temperature of the silicone oil bath was gradually decreased to RT at a rate of 1 ℃/h. Once the cooling process was completed, large orange plates of $BA_2PbI_4$ were seen in the bottom of the vial. The single crystals were then taken out by evacuating the supernatant and drying them gently and thoroughly with fiber-free blotting paper. The crystals were then evacuated overnight in the anti-chamber of a glove box and stored in a $N_2$-filled glovebox until further measurements were performed. For the SPV measurements, the single crystals were mounted on ITO substrates using EPOTEK H20E silver epoxy. For the CLIMAT measurements, the crystals were mounted using EPOTEK H20E silver epoxy on glass substrates with patterned Cr/Au contacts.

### *XRD*

XRD characterization of C4N1 single crystals was carried out in reflection geometry using Rigaku (Tokyo, Japan) theta-theta diffractometer, TTRAX III, equipped with a rotating copper anode X-ray tube operating at 50 kV/200 mA. A scintillation detector was aligned to intersect the diffracted beam after it passed a bent graphite monochromator to remove Kβ radiation. Using open slits after a sample that was rotating about its normal, a $\theta - 2\theta$ scan was performed from 1.5° −30° $2\theta$ with a step size of 0.01° and a scan rate of 5° per minute. The XRD results indicate that no impurity phases are present, as well as a uniform <100> orientation of the crystal.

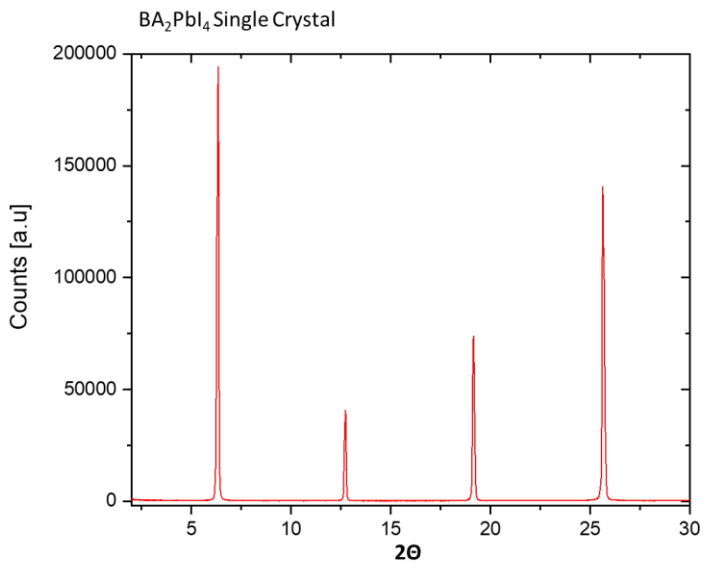


Figure S18 –XRD of the single crystal

*Polycrystalline thin film preparation*

Patterned indium tin oxide (ITO) glass substrates were sequentially cleaned using Hellmanex solution 1% vol., water, Acetone, and isopropanol in an ultrasonic bath. After cleaning, the substrates were treated in a UV-ozone cleaner for 15 minutes.

To prepare the $A_2PbX_4$ (A = BA / PEA; X = I/Br) perovskite precursor solution, $PbX_2$ (TCI) and AI (Sigma-Aldrich) were dissolved stoichiometrically in DMF to a nominal concentration of 0.5M by shaking overnight at 60°C. The perovskite solution was deposited on top of the ITO by spin-coating at 5000 rpm (2 s acceleration) for 50s without using an antisolvent. After the spin-coating program, the perovskite-coated sample was annealed at 100°C for 20 min on a hotplate. The perovskite fabrication process was carried out in a N2 filled glovebox.

*PL measurements*

PL measurements were carried out on a home-built confocal PL setup utilizing a 90:10 transmission:reflection beamsplitter to separate the excitation and detection paths. 472 nm photoexcitation was supplied by a SuperK Fianium supercontinuum laser with a Varia filter unit equipped (NKT Photonics) at a repetition rate of 72 MHz. The Varia bandwidth was 10nm.

Focusing and PL collection was by an off-axis parabolic mirror with 5cm focal length, the laser spot was a circle with a 1/e diameter of approximately 35µm. Photoluminescence detection was by a USB4000 spectrometer (Ocean Optics) with 5s integration time. A 480nm long pass filter was used in the detection path. Laser power was 0.2mW at the sample.

*Modulated SPV spectroscopy*

Modulated SPV spectra were measured in the configuration of a parallel plate capacitor (quartz cylinder partially coated with the SnO2:F electrode, cover glass #0 as insulator).[2] The Illumination was provided by a Halogen lamp, coupled to a quartz prism monochromator (SPM2), and modulated at a frequency of 8 Hz by using an optical chopper. In-phase and 90$^{o}$ phase-shifted SPV signals were detected with a high-impedance buffer and a double phase lock-in amplifier (EG&G 5210).

*Transient SPV spectroscopy*

Transient SPV spectroscopy measurements were performed using an oscilloscope card (Gage, CSE 1622-4GS), a tunable Nd:YAG laser for excitation (duration time of laser pulses 3–5 ns, range of wavelengths 216–2600 nm (EKSPLA, NT230–50, equipped with a spectral cleaning unit), and a tunable beam expander to obtain a relatively constant photon flux across the excitation wavelength range (as shown in figure S4). The repetition rate of the laser pulses was 2 Hz, and 20 transients were averaged. Two different SPV electrodes were used: for the measurement under a constant photon flux (Figure 4 in the main text), a fixed perforated electrode with a charge amplifier (Electronik Manufaktur Mahlsdorf, resolution time 7 ns) was used. Other measurements (Figures 5 and S6) were performed in the configuration of a parallel plate capacitor (quartz cylinder partially coated with the SnO2:F electrode, cover glass #0 as an insulator) with a high-impedance buffer.[14]

To confirm that the observed onset of defect-related SPV signals for the single crystal in the tr-SPV measurements at 1.2 eV does not originate from experimental artefacts such as variations in the laser intensity as a function of wavelength (which was kept nearly constant, as shown in Figure S4) or processes related to 2-photon absorption (due to the high laser power used in the tr-SPV measurement), sub-band gap modulated SPV spectra was recorded, and a similar onset was observed, as shown in Figure S2

*Constant light-induced magneto-transport (CLIMAT)*

CLIMAT measurements were performed by using an AC magnetic field with a lock-in amplifier. The amplifier is used to improve the Hall effect signal because of the low mobility and conductivity of the metal halide perovskite materials. The encapsulated perovskite samples were characterized by using a four-point-probe (Van Der Pauw) in He atmosphere at room temperature. A 0.6 T magnetic field amplitude with 100 Hz frequency was introduced for the CLIMAT measurement. Additionally, a LED with an emission energy 1.5 eV, 2.0 eV, and 2.8 eV were employed for generating the free carrier of perovskite thin films.

The intensity dependent characterization of the charge transport constants was measured up to one sun equivalent power (100 mW/cm$^2$). In particular, the Longitudinal voltage and Hall voltage were measured, from which the conductivity, Hall coefficient, and Hall mobility were calculated. More details on the method can be found in our previous publication.[15] The samples were encapsulated under nitrogen atmosphere and stored in the nitrogen-filled glovebox before measurements.

*Constant Final State Yield Spectroscopy (CFSYS)*

Sample preparation: To avoid charging during the measurements, the single crystal was mounted and glued on ITO-covered glass substrate using a 2-component silver epoxy (EPOTEK H20E). Both the single crystal and the thin film underwent inert transfer directly to the UHV chamber from an $N_2$-filled glovebox (the single crystal was peeled with a 3M scotch tape inside the glovebox prior to the transfer).

CFSYS measurements: The samples were electrically grounded by two metal contacts, which were pressed onto the ITO on the sample edge, to ensure a well-working electrical contact. First, a near-UV photoelectron spectroscopy (PES) measurement was performed at a fixed excitation energy of 6.5 eV, to determine the kinetic energy that yields the highest count rate, followed by the CFSYS measurement. In CFSYS collection mode, photoelectrons at one constant final state (one kinetic energy, determined from the near-UV PES measurement) are detected, while the photon (excitation) energy is varied. Xenon short arc lamp (XBO) was used in combination with a double grating monochromator to yield photon energies between 3 eV and 7 eV. For more details please see our previous study.[1]

## References


[1] D. Menzel, A. Tejada, A. Al-Ashouri, I. Levine, J.A. Guerra, B. Rech, S. Albrecht, and L. Korte, "Revisiting the Determination of the Valence Band Maximum and Defect Formation in Halide Perovskites for Solar Cells: Insights from Highly Sensitive Near–UV Photoemission Spectroscopy," ACS Appl. Mater. Interfaces **13**(36), 43540–43553 (2021).

[2] C.M. Perez, D. Ghosh, O. Prezhdo, S. Tretiak, and A.J. Neukirch, "Excited-State Properties of Defected Halide Perovskite Quantum Dots: Insights from Computation," J. Phys. Chem. Lett. **12**(3), 1005–1011 (2021).

[3] P. Hohenberg, and W. Kohn, "Inhomogeneous electron gas," Phys. Rev. **136**(3B), B864 (1964).

[4] W. Kohn, and L.J. Sham, "Self-consistent equations including exchange and correlation effects," Phys. Rev. **140**(4A), A1133 (1965).

[5] G. Kresse, and D. Joubert, "From ultrasoft pseudopotentials to the projector augmented-wave method," Phys. Rev. B **59**(3), 1758 (1999).

[6] P.E. Blöchl, "Projector augmented-wave method," Phys. Rev. B **50**(24), 17953 (1994).

[7] J.P. Perdew, and K. Burke, "Generalized gradient approximation for the exchange-correlation hole of a many-electron system," Phys. Rev. B **54**(23), 16533 (1996).

[8] J.P. Perdew, K. Burke, and M. Ernzerhof, "Generalized Gradient Approximation Made Simple," Phys. Rev. Lett. **77**(18), 3865 (1996).

[9] G. Kresse, and J. Hafner, "*Ab initio* molecular dynamics for liquid metals," Phys. Rev. B **47**(1), 558 (1993).

[10] G. Kresse, and J. Furthmüller, "Efficiency of ab-initio total energy calculations for metals and

semiconductors using a plane-wave basis set," Comput. Mater. Sci. **6**(1), 15–50 (1996).

[11] G. Kresse, and J. Hafner, "*Ab initio* molecular-dynamics simulation of the liquid-metal–amorphous-semiconductor transition in germanium," Phys. Rev. B **49**(20), 14251 (1994).

[12] G. Kresse, and J. Furthmüller, "Efficient iterative schemes for *ab initio* total-energy calculations using a plane-wave basis set," Phys. Rev. B **54**(16), 11169 (1996).

[13] C.C. Stoumpos, D.H. Cao, D.J. Clark, J. Young, J.M. Rondinelli, J.I. Jang, J.T. Hupp, and M.G. Kanatzidis, "Ruddlesden-Popper Hybrid Lead Iodide Perovskite 2D Homologous Semiconductors," Chem. Mater. **28**(8), 2852–2867 (2016).

[14] T. Dittrich, S. Fengler, and M. Franke, "Transient surface photovoltage measurement over 12 orders of magnitude in time," Rev. Sci. Instrum. **88**(5), 053904 (2017).

[15] A. Musiienko, F. Yang, T.W. Gries, C. Frasca, D. Friedrich, A. Al-Ashouri, E. Sağlamkaya, F. Lang, D. Kojda, Y.T. Huang, V. Stacchini, R.L.Z. Hoye, M. Ahmadi, A. Kanak, and A. Abate, "Resolving electron and hole transport properties in semiconductor materials by constant light-induced magneto transport," Nat. Commun. 2024 151 **15**(1), 1–11 (2024).
